# An integrated transcriptomic and metabolomic analysis of black spot disease in Jinggang honey pomelo reveals underlying resistance mechanisms

**DOI:** 10.3389/fmicb.2025.1495804

**Published:** 2025-05-15

**Authors:** Huimin Sun, Weiwei Li, Lin Lu, Biling Jin, Dingkun Liu, Zexia Li, Li He, Yang Wu

**Affiliations:** School of Life Sciences, Jinggangshan University, Ji'an, China

**Keywords:** Jinggang honey pomelo, black spot disease, *D. citri*, transcriptome, metabolome

## Abstract

**Introduction:**

The Jinggang honey pomelo is recognized as one of the three major fruit industry brands in Jiangxi Province. However, the crop's growth and yield have been significantly affected by the black spot disease caused by *Diaporthe citri*. Despite this impact, the defense mechanisms and underlying molecular responses of the Jinggang honey pomelo to the disease remain poorly understood.

**Methods:**

In this study, we utilized UPLC-MS/MS and RNA-Seq to conduct a comparative analysis of differentially abundant metabolites (DAMs) and differentially expressed genes (DEGs) in uninfected and *D. citri*-infected Jinggang honey pomelo fruits 13 days post-infection (dpi) *in vivo*.

**Results:**

Our analysis yielded 1,744, 1,616, and 1,325 DAMs, as well as 3,403, 1,767, and 453 DEGs from the respective varieties, with 426 DAMs and 66 DEGs common across all three. Kyoto Encyclopedia of Genes and Genomes enrichment analysis demonstrated significant enrichment of these DAMs and DEGs in phenylpropanoid and flavonoid biosynthesis pathways. We also discovered that transcription factors (TFs), specifically *MYB* and *bHLH*, related to these pathways, were highly expressed. Our elucidation of the phenylpropanoid and flavonoid biosynthesis pathways surmises that genes (*4CL, F5H, HCT, CCR*, and *CAD*) and metabolites (p-coumaryl acetate, pinocembrin, naringin, and neohesperidin) could significantly contribute to the resistivity of Jinggang honey pomelo against *D. citri*.

**Discussion:**

Our findings suggest that Jinggang honey pomelo activates phenylpropanoid and flavonoid biosynthesis pathways, leading to the accumulation of flavonoid compounds that resist *D. citri* invasion. This study lays the groundwork for further research into the molecular mechanisms and breeding of Jinggang honey pomelo resistant to black spot disease.

## 1 Introduction

The Jinggang honey pomelo, also known as the “Jinlan pomelo,” “Jinsha pomelo,” and “Taoxi pomelo,” is a premium-quality fruit cultivated in Ji'an City, Jiangxi Province. The Jinggang honey pomelo is thin-skinned with few seeds and is rich in vitamins B1 and B2. It has notably high levels of vitamin C, calcium, and magnesium compared to other pomelos. This fruit is beneficial for regulating metabolism, assisting digestion, promoting weight loss, and enhancing skin health. It is also known to alleviate conditions such as hypertension and diabetes, earning it the name “King of Pomelos.” The Jiangxi Provincial Department of Agriculture has recognized the Jinggang honey pomelo as one of the three major fruit industry brands in Jiangxi, actively promoting it for further recognition and appreciation.

Black spot disease in Jinggang honey pomelo is a form of citrus black spot disease. This significant fungal disease, as stated by Fang et al. (2022) and Ying et al. (2017), has been prevalent in Shanghai (Fei et al., 2022), Hunan (Yang et al., 2021), Zhejiang (Weigen et al., 2019), and other regions. Moreover, black spot disease is among the most severe diseases in the tropical and subtropical citrus-growing regions of Africa, Asia, Oceania, and the Americas (Silva-Junior et al., 2016; Kotzé, 2000; Fernandes et al., 2022). *D. citri* is one of the most destructive fungal pathogens of citrus (Li-ying et al., 2012; Guarnaccia and Crous, 2017). It infects young leaves, shoots, and fruits, producing black-to-reddish brown, raised pustules (known as melanose) on the leaves, twigs, and fruits of citrus (Nelson, 2008). Typically, although melanose does not decrease yield, it affects the marketability of citrus fruits, resulting in significant economic losses (Li-ying et al., 2012; Rehman et al., 2020). *D. citri* can also induce stem-end rot, shoot-blight and dieback, trunk or branch gummosis, and rot in all citrus species or varieties worldwide (Li-ying et al., 2012; Guarnaccia and Crous, 2017; Guoqing, 2010; Huang et al., 2013; Fawcett, 1912). When *D. citri* conidia contact the host leaf surface, they germinate, forming appressoria to penetrate the cuticle and extend hyphae into the mesophyll tissue. This results in cell lysis and exudation of a gelatinous substance that hardens into dark protuberances (Bach and Wolf, 1929; Gopal et al., 2014). Additionally, *D. citri* pectinase, during fruit maturation or storage, can promote fruit rot symptoms (Bach and Wolf, 1929; Prusky et al., 2013; Bahgat, 1928).

Diseases emerge when pathogens overcome the plant's immune system (Jones and Dangl, 2006). In plant-pathogen interactions, pathogens redirect host nutrients for their survival and reproduction, while plants employ defenses to inhibit pathogen growth (Ngou et al., 2022). Multi-omics techniques, such as genomics, transcriptomics, proteomics, and metabolomics, can differentiate between plants with and without black spot disease treatment. Previous studies have preliminarily identified defense-related genes in citrus black spot disease. Li et al. (2022) demonstrated that *Phomopsis citri* infection significantly reduces the evenness of citrus leaf microbiomes but increases populations of antagonistic bacteria Pantoea asv90 and Methylobacterium asv41, which is potentially linked to the citrus immune response. Previous studies using microscopy and HPLC analysis have shown that citrus leaves trigger defense responses upon *P. citri* infection, including the induction of the defense compound 6,7-dimethoxy coumarin, which limits pathogen spread (Arimoto et al., 1986a, 1982, 1986b). Li et al. (2023) used RNA-Seq to analyze the transcriptomes of citrus leaves at 3 and 14 days post-infection with the greasy spot pathogen, finding that cell wall biogenesis genes were prominently induced at 3 days, while genes implicated in suberin deposition, pectin methylesterase, and coumarin synthesis (e.g., Feruloyl-CoA 6′-Hydroxylase1 and scopoletin 8-hydroxylase) were significantly induced at 14 days.

While previous studies have granted preliminary insights into the molecular mechanisms of citrus black spots using singular-omics approaches, these methods are hampered by incomplete information, challenges in revealing mechanisms, and inadequate functional annotations. Considering that plant responses to pathogen infection involve large-scale changes in gene expression and metabolism, merging transcriptomics and metabolomics presents a potent approach for a comprehensive comprehension of plant defense mechanisms at the molecular and cellular levels (Li et al., 2021).

In this study, transcriptomic and metabolomic analyses were conducted to examine the transcriptional and metabolic shifts in Jinggang honey pomelo in response to black spot infection. This work lays the groundwork for breeding and enhancing resistant varieties, clarifying resistance mechanisms, and facilitating integrated antifungal omics research in Jinggang honey pomelo.

## 2 Materials and methods

### 2.1 Sample preparation

Eight-year-old pomelo trees of the Jinlan (af), Jinsha (jsh), and Taoxi (qy) varieties, located in a greenhouse with a 30/18°C day/night temperature regime and natural sunlight at the Ji'an Agricultural High-tech Industry Demonstration Zone in Jiangxi Province, were selected as experimental samples. The surface of every pomelo fruit was disinfected with 75% ethanol and then rinsed with sterile water. The *D. citri* isolate used was sourced from diseased pomelo plants and cultivated in Potato Dextrose Agar (PDA) medium at 26°C. After 7 days, a mycelial plug measuring 0.6 cm in diameter was inoculated onto the fruit surface employing a puncture inoculation method. The control group received an inoculation with a sterile PDA medium. Following inoculation, each plant was housed in a clear plastic bag, and sprinkled with water, to maintain high humidity for 3 days. Changes in the fruit epidermis were assessed post-inoculation, and honey pomelo fruit samples were harvested 13 days after inoculation. These samples were instantly frozen in liquid nitrogen and preserved at −80°C for subsequent metabolome and transcriptome analysis. Each treatment encompassed three biological replicates.

### 2.2 Metabolomics analysis

#### 2.2.1 Metabolites extraction and machine testing

Approximately 50 mg of each sample was weighed and extracted with 1,000 μL of solvent containing an internal standard (20 mg/L), prepared with a methanol:acetonitrile:water ratio of 2:2:1 (v/v/v). The mixture was vortexed for 30 s to ensure thorough mixing. Steel beads were added, and the sample was homogenized using a grinding instrument at 45 Hz for 10 min. This was followed by sonication in an ice-water bath for 10 min. Samples were then incubated at −20 °C for 1 hand subsequently centrifuged at 12,000 rpm at 4 °C for 15 min. A volume of 500 μL of the resulting supernatant was carefully transferred to an EP tube, avoiding disturbance of the pellet, and dried using a vacuum concentrator.

The dried extract was reconstituted in 160 μL of extraction solvent (acetonitrile:water, 1:1, v/v), vortexed for 30 s, and sonicated again in an ice-water bath for 10 min. The samples were centrifuged once more at 12,000 rpm at 4 °C for 15 min. Finally, 120 μL of the supernatant was transferred into a 2 mL vial for analysis. To prepare a quality control (QC) sample, 10 μL was pooled from each individual sample. The LC/MS system utilized for metabolomics analysis comprises a Waters Acquity I-Class PLUS ultra-high-performance liquid chromatograph, which is coupled with a Waters Xevo G2-XS QTOF high-resolution mass spectrometer. The column employed is a Waters Acquity UPLC HSS T3 column (1.8 μm, 2.1 × 100 mm).

In positive ion mode, mobile phase A is comprised of a 0.1% formic acid aqueous solution, while mobile phase B contains 0.1% formic acid in acetonitrile. For the negative ion mode, mobile phases A and B remain identical to those in the positive ion mode. The volume set for injection is 1 μL.

#### 2.2.2 LC-MS/MS analysis

The Waters Xevo G2-XS QTOF high-resolution mass spectrometer can collect both primary and secondary mass spectrometry data in MSe mode, under the control of acquisition software (MassLynx V4.2, Waters). In each data acquisition cycle, it can simultaneously perform dual-channel data acquisition for both low and high collision energies. The low collision energy is 2 V, while the high collision energy range is between 10 and 40 V, and the scanning frequency is 0.2 s for a mass spectrum. The parameters of the ESI ion source include: a capillary voltage of 2,000 V in positive ion mode or −1,500 V in negative ion mode, cone voltage of 30 V, ion source temperature of 150°C, desolvent gas temperature of 500°C, a backflush gas flow rate of 50 L/h, and a desolventizing gas flow rate of 800 L/h.

#### 2.2.3 Data preprocessing and annotation

The raw data collected using MassLynx V4.2 was processed using the Progenesis QI software. This software handles peak extraction, peak alignment, and other data processing operations, referencing the Progenesis QI software's online METLIN database (https://ngdc.cncb.ac.cn/databasecommons/database/id/5907) and Biomark's self-constructed library for identification. Simultaneously, theoretical fragment identification and mass deviation are all maintained within 100 ppm.

#### 2.2.4 Data analysis

After normalizing the initial peak area information with the total peak area, we conducted subsequent analyses. We used principal component analysis and Spearman correlation analysis to evaluate the reproducibility of the samples within each group, as well as the QC samples. We sourced classification and pathway information for the identified compounds in the Kyoto Encyclopedia of Genes and Genomes (KEGG) (http://www.genome.jp/kegg/), HMDB (https://hmdb.ca/), and Lipid Maps (https://lipidmaps.org/) databases. We calculated and compared fold changes according to the group information, and used a *t*-test to compute the significance *p*-values of the differences for each compound. We employed the R language package “ropls” for OPLS-DA modeling, and conducted 200 permutation tests to confirm the model's reliability. We calculated the model's VIP value through multiple cross-validation. By using a method that combined fold changes, *p-values, and the VIP value from the OPLS-DA model, we were able to screen diffe*rential metabolites. The screening criteria are a fold change >1, *P*-value < 0.05, and VIP >1. We determined the significance of KEGG pathway enrichment for differential metabolites using a hypergeometric distribution test.

### 2.3 Transcriptome analysis

#### 2.3.1 RNA extraction

Plant total RNA was extracted using the RNAprep Pure Plant Kit (Tiangen, Beijing, China), following the manufacturer's instructions. Similarly, total RNA was obtained as per the instruction manual provided with the TRIzol Reagent (Life Technologies, California, USA).

#### 2.3.2 Library construction and sequencing

The concentration and purity of RNA were measured using the NanoDrop 2000 (Thermo Fisher Scientific, Wilmington, DE). The integrity of the RNA was assessed using the RNA Nano 6000 Assay Kit on the Agilent Bioanalyzer 2100 system (Agilent Technologies, CA, USA).

Sequencing libraries were generated using the Hieff NGS Ultima Dual-mode mRNA Library Prep Kit for Illumina (Yeasen Biotechnology (Shanghai) Co., Ltd.), according to the manufacturer's recommendations, and index codes were applied to assign sequences to particular samples. Briefly, mRNA was isolated from total RNA using poly-T oligo-attached magnetic beads. First-strand cDNA synthesis was conducted, followed by second-strand cDNA synthesis. Any remaining overhangs were transformed into blunt ends using exonuclease and polymerase activities. After the adenylation of the 3′ ends of cDNA fragments, NEBNext Adaptors with hairpin loop structures were ligated. The library fragments were then purified with the AMPure XP system (Beckman Coulter, Beverly, USA). Next, 3 μL of USER Enzyme (NEB, USA) was used with size-selected, adaptor-ligated cDNA at 37°C for 15 min, followed by 5 min at 95°C preceding PCR. PCR was subsequently performed using Phusion High-Fidelity DNA polymerase, Universal PCR primers, and Index (X) primers. Lastly, PCR products were purified with the AMPure XP system, and the library quality was assessed using the Agilent Bioanalyzer 2100 system.

The libraries were sequenced on an Illumina NovaSeq platform to produce 150 bp paired-end reads, following the manufacturer's instructions.

#### 2.3.3 Transcriptome assemble

The raw reads underwent further processing using a bioinformatics pipeline tool, namely, the BMKCloud (http://www.biocloud.net) online platform. The raw reads, in fastq format, were initially processed using in-house Perl scripts. During this process, clean reads were secured by eliminating adaptor sequences, reads containing poly-N, and low-quality reads from the raw data. Additionally, the clean data's Q30, GC content, and sequence duplication level were calculated. All downstream analyses were based on high-quality clean data.

These clean reads were subsequently mapped to the reference genome sequence. Only reads with an identical match or one mismatch were further examined and annotated based on the reference genome. The HISAT2 (https://daehwankimlab.github.io/hisat2/) was utilized to map to the reference genome. By utilizing the HISAT2 software, clean reads were aligned swiftly and accurately with the *Citrus maxima* reference genome. Lastly, the StringTie (https://ccb.jhu.edu/software/stringtie/) Reference Annotation-Based Transcript assembly method was employed to construct and identify both known and novel transcripts from the HISAT2 alignment results.

#### 2.3.4 Genes annotation

Gene functions were annotated using the following databases: Nr (NCBI non-redundant protein sequence database) (ftp://ftp.ncbi.nih.gov/blast/db/; Pfam (protein family) (http://pfam.xfam.org/); KOG (Clusters of Protein homology) (http://www.ncbi.nlm.nih.gov/KOG/); COG (Clusters of Orthologous Groups of proteins) (http://www.ncbi.nlm.nih.gov/COG/); Swiss-Prot (a manually annotated and reviewed protein sequence database) (http://www.uniprot.org/); KO (KEGG Ortholog database) (http://www.genome.jp/kegg/); GO (Gene Ontology) (http://www.geneontology.org/).

#### 2.3.5 Quantification of gene expression levels

Gene expression levels were quantified using fragments per kilobase of transcript per million mapped fragments. The formula is as follows: FPKM = cDNA Fragments/[Mapped Fragments (in millions) × Transcript Length (in kilobases)].

#### 2.3.6 Differential expression analysis

For the samples with biological replicates, the differential expression analysis of two conditions/groups was conducted using DESeq2 (https://bioconductor.org/packages/release/bioc/html/DESeq2.html). DESeq2 provides statistical routines for determining differential expression in digital gene expression data, using a model based on the negative binomial distribution. The resulting *p*-values were adjusted using Benjamini and Hochberg's method for controlling the false discovery rate. Genes with an adjusted P-value of less than 0.01 and a fold change equal to or greater than 1.5, as identified by DESeq2, were categorized as differentially expressed.

#### 2.3.7 GO enrichment analysis

The GO enrichment analysis of the differentially expressed genes (DEGs) was performed using the clusterProfiler R package.

#### 2.3.8 KEGG pathway enrichment analysis

We utilized the KOBAS (https://www.biostars.org/p/300733/) database and the clusterProfiler software to test the statistical enrichment of DEGs in KEGG pathways.

#### 2.3.9 Transcription factors prediction

The gene sets that were differentially expressed were used as the candidate genes. We predicted transcription factors (TFs) using the TFDB database with hmmsearch for both animal and human data.

### 2.4 Correlation analysis of the transcriptome and metabolome datasets

A joint analysis of the DEGs and differentially abundant features (DAFs) was conducted to ascertain the level of pathway enrichment via a correlation heat map, a correlation matrix, and an association network diagram. Pathway information shared by the DEGs and DAFs was projected onto GO and KEGG.

## 3 Results

### 3.1 Phenotypes of three jinggang honey pomelo after infection by *D. citri*

**Figure 1:**
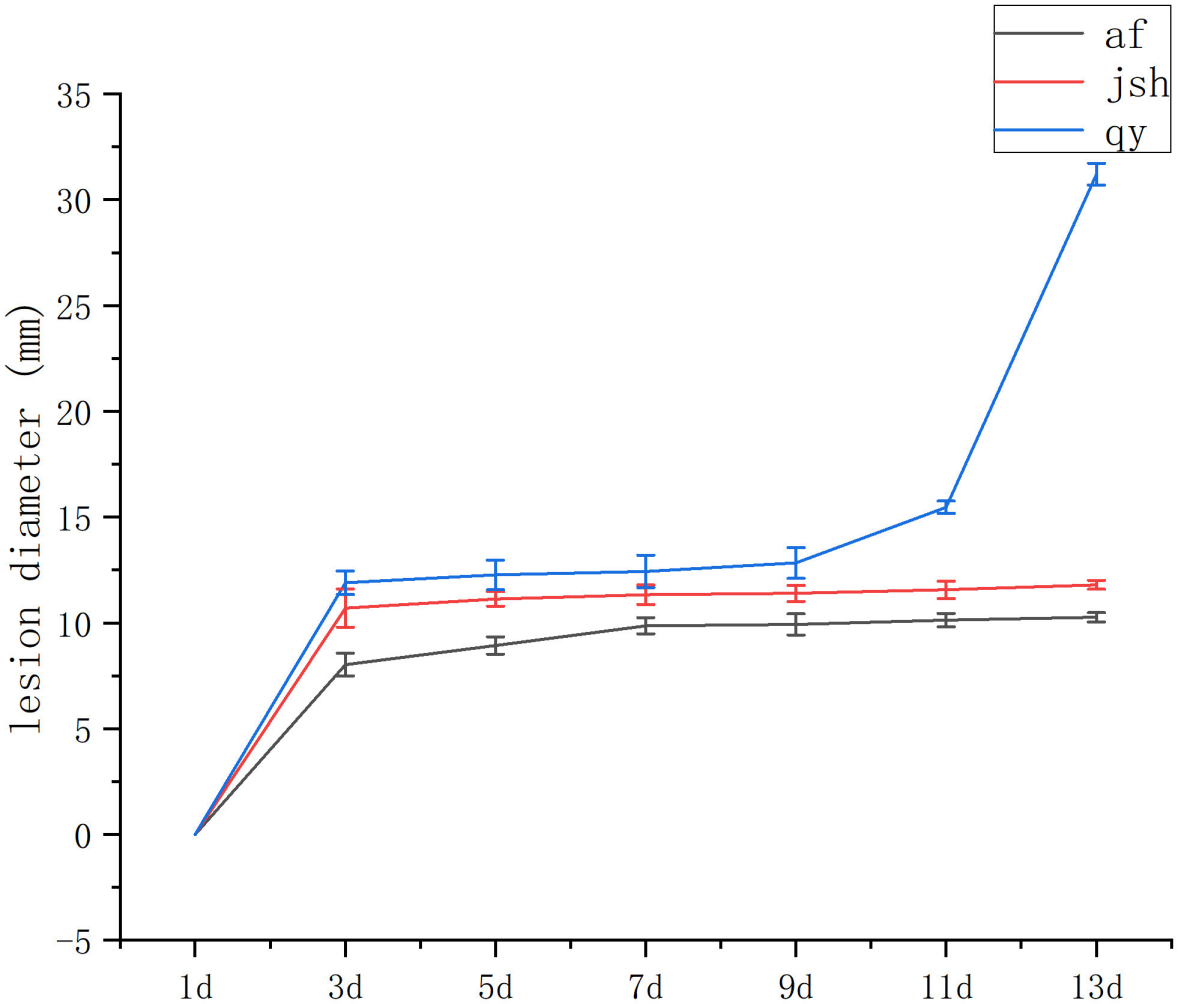
Fruit lesion size over time of af, jsh and qy after inoculation with *D. citri, n* = 3.

Our recent field study of cultivated accessions revealed that the individuals af, jsh, and qy exhibited various degrees of resistance to black spot disease. Af and jsh were more resistant to black spot disease. To verify the results of the field survey, *D. citri* was inoculated onto the fruit of af, jsh, and qy. Infected fruits from these three lines showed obvious symptoms of black spot disease (Supplementary Figure 1). The diameters of the lesions were measured during disease progression. At 13 days post-inoculation with *D. citri*, the lesion diameter of qy was significantly larger than that of af and jsh (Figure 1). Based on the aforementioned results, af and jsh were identified as resistant cultivars, while qy was identified as a susceptible cultivar.

### 3.2 Analysis of Jinggang honey pomelo metabolome in response to *D. citri*

A fungal disc, 6 mm in diameter, was used to inoculate Jinggang honey pomelo fruit to investigate the fruit's metabolic response to *D. citri* infection. After 13 days, both healthy (uninoculated, designated as afCK for af, jshCK for jsh, and qyCK for qy) and diseased (inoculated, designated as afHD for af, jshHD for jsh, and qyHD for qy) fruits were gathered for metabolomic analysis.

**Figure 2:**
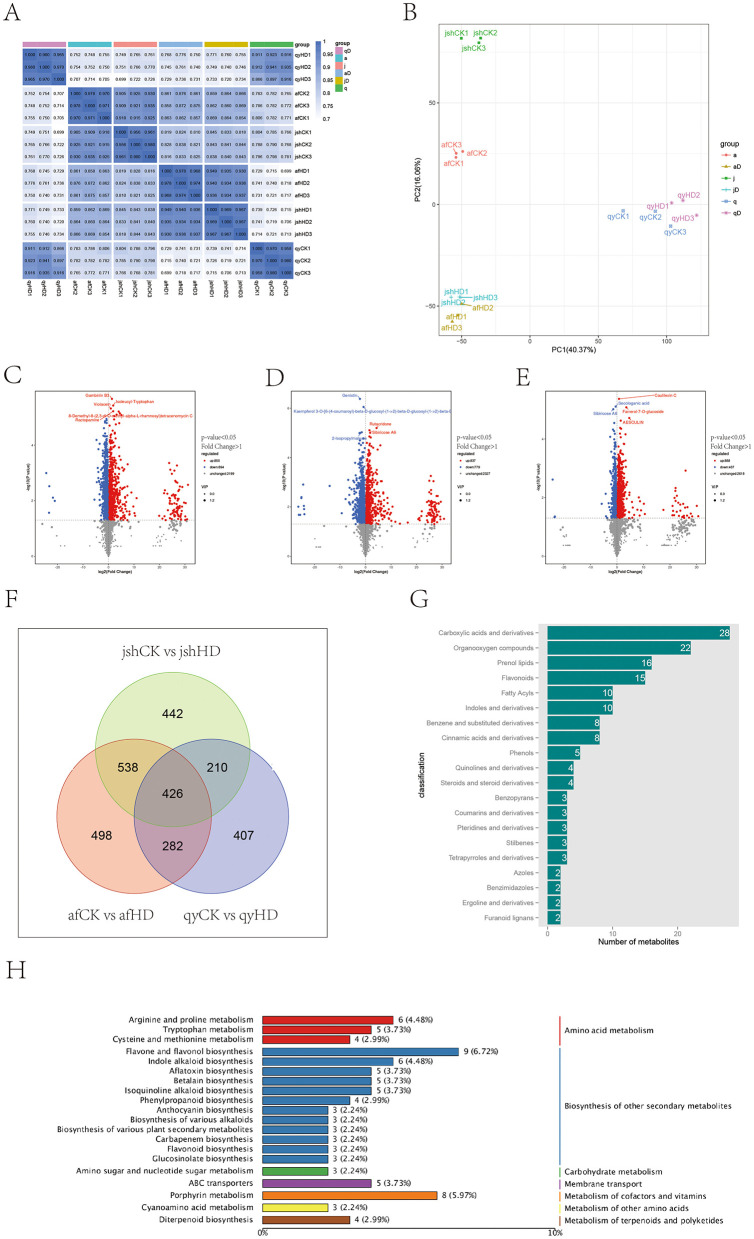
Analysis of Jinggang honey pomelo metabolome in response to *D. citri*. **(A)** Sample correlation analysis; **(B)** PCA score plot; **(C–E)** Number of up/down-regulated DAMs between the three groups (afCK vs. afHD, jshCK vs. jshHD, and qyCK vs. qyHD); **(F)** Venn diagram showing DAMs shared among afCK vs. afHD, jshCK vs. jshHD, and qyCK vs. qyHD; **(G)** Classification of the common DAMs; **(H)** KEGG analysis of the common DAMs.

Through the analysis of the correlation between samples, we utilized the Spearman Rank Correlation coefficient (*r*) as a metric for evaluating the correlation of biological replicates. The closer the *r* value is to 1, the greater the correlation between the replicate samples. The results suggested that reproducibility among samples within each group was satisfactory, with *r* values all exceeding 0.95 (Figure 2A). Further analysis via principal component analysis displayed a significant separation between qyCK vs. qyHD, afCK vs. afHD, and jshCK vs. jshHD, accounting for the first principal component, at 40.37% (Figure 2B). Moreover, samples from afCK vs. afHD and jshCK vs. jshHD were distinctly differentiated on the second principal component at 16.06%. These findings imply that the results from the metabolomic test are appropriate for subsequent differential metabolite screening analysis.

By employing both univariate and multivariate statistical analysis methods, a total of 3,943 metabolites were pinpointed across the samples of afCK vs. afHD, jshCK vs. jshHD, and qyCK vs. qyHD. The identification of differentially abundant metabolites (DAMs) was based on a VIP score of ≥1 and a *P*-value of < 0.05, resulting in 1,744, 1,616, and 1,325 DAMs, respectively. Of these, the numbers of upregulated DAMs counted 850, 837, and 888, while the numbers of downregulated DAMs totaled 894, 779, and 437, respectively (Figures 2C–E).

By examining the common DAMs in different resistance levels of Jinggang honey pomelo, we can achieve a profound understanding of the physiological and biochemical response mechanisms of Jinggang honey pomelo in response to *D. citri* infection. These DAMs may serve as key defensive substances or energy metabolism products that play a crucial role in the resistance response of Jinggang honey pomelo. The Venn diagram shows that there are 426 common DAMs across the three sample groups (Figure 2F). Among these 426 common DAMs, 211 were upregulated in afCK vs. afHD, 387 in jshCK vs. jshHD, and 309 in qyCK vs. qyHD, while 215 were downregulated in afCK vs. afHD, 239 in jshCK vs. jshHD, and 117 in qyCK vs. qyHD (Supplementary Table 1). The classification of the common DAMs includes carboxylic acids and their derivatives, organic oxygen compounds, isoprenoids, flavonoids, fatty acids, indoles and their derivatives, benzene, and its substituted derivatives, cinnamic acid, and its derivatives, and phenols, among others (Figure 2G).

KEGG enrichment analysis primarily revealed an enrichment of common DAMs in the biosynthesis of secondary metabolites. Among these, the biosynthesis pathways of flavonoid compounds, including flavone and flavonol biosynthesis, phenylpropanoid biosynthesis, anthocyanin biosynthesis, and flavonoid biosynthesis, along with alkaloid biosynthesis, such as indole alkaloid biosynthesis, isoquinoline alkaloid biosynthesis, and the biosynthesis of various alkaloids, were notably enriched (Figure 2H). These pathways may represent the main routes through which af, jsh, and qy engage in the stress response to *D. citri*.

**Figure 3:**
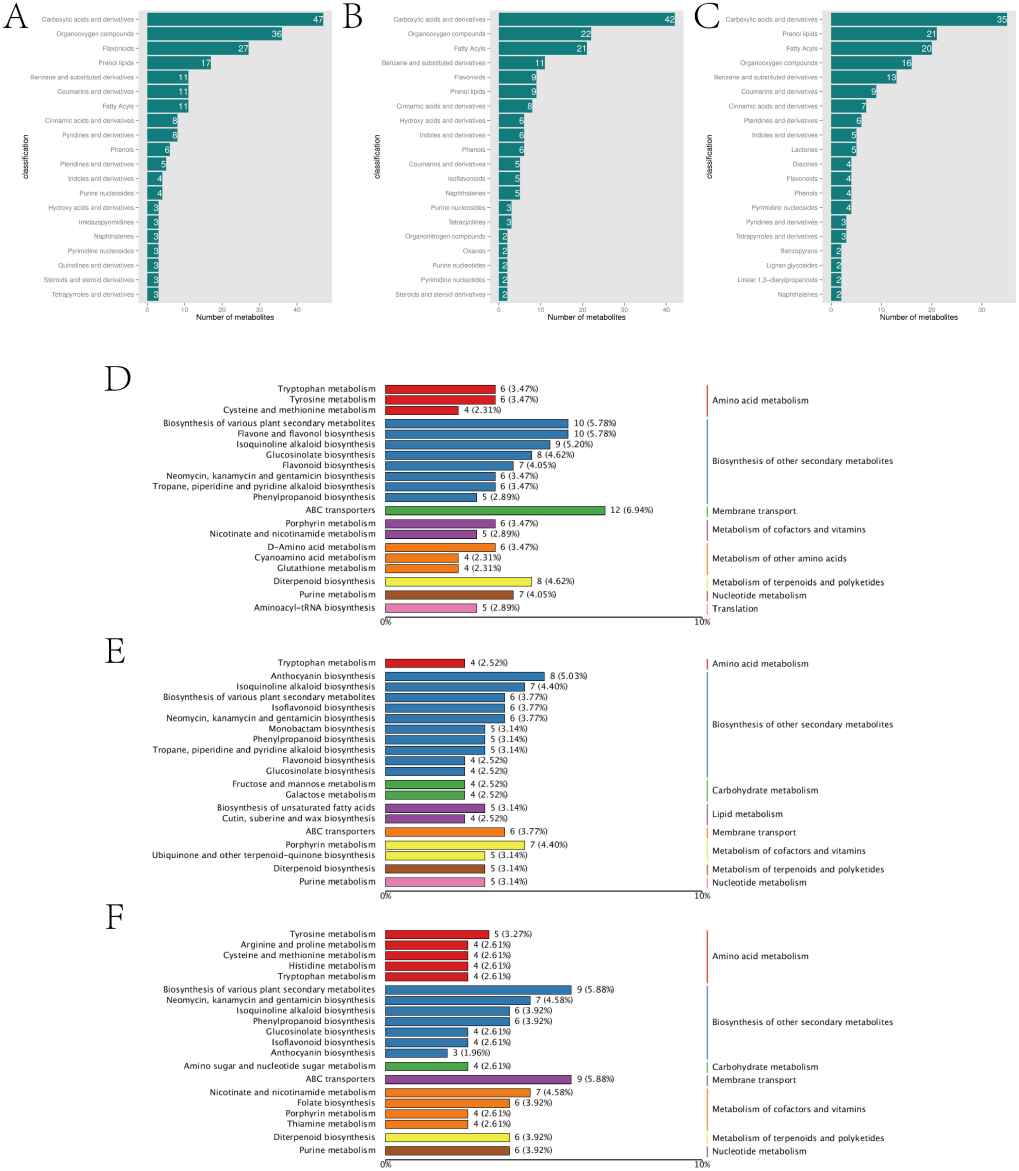
Analysis of Jinggang honey pomelo metabolome in response to *D. citri*. **(A)** Classification of the specific DAMs in afCK vs. afHD; **(B)** Classification of the specific DAMs in jshCK vs. jshHD; **(C)** Classification of the specific DAMs in qyCK vs. qyHD; **(D)** KEGG analysis of the specific DAMs to afCK vs. afHD; **(E)** KEGG analysis of the specific DAMs to jshCK vs. jshHD; **(F)** KEGG analysis of the specific DAMs to qyCK vs. qyHD.

In addition to 426 common DAMs, the comparisons of afCK vs. afHD, jshCK vs. jshHD, and qyCK vs. qyHD each produced 498, 442, and 407 specific DAMs, respectively. Notably, the afCK vs. afHD and jshCK vs. jshHD samples contained 27 and 14 flavonoid compounds (flavonoids and isoflavonoids), respectively, while the qyCK vs. qyHD sample contained only four (Figures 3A–C). Flavonoids play multiple roles in plant disease resistance, including acting as antioxidants, inhibiting pathogen growth, inducing plant defense responses, enhancing the defensive capacity of plant cell walls, modulating plant hormone signaling, and serving as precursors for plant volatile organic compounds. These mechanisms work synergistically to enhance the resistance of plants to pathogens (Dixon, 2001).

The specific DAMs in afCK vs. afHD, jshCK vs. jshHD, and qyCK vs. qyHD were primarily enriched in the biosynthesis of secondary metabolites. The specific DAMs in afCK vs. afHD and jshCK vs. jshHD were enriched mainly in flavone and flavonol biosynthesis, and anthocyanin biosynthesis, respectively. The specific DAMs in qyCK vs. qyHD were primarily enriched in neomycin, kanamycin, and gentamicin biosynthesis (Figures 3D–F). The black spot disease of the Jinggang honey pomelo, caused by *D. citri*, is a fungal disease. Flavones, flavonols, and anthocyanins, the three major subclasses of flavonoids, display significant inhibitory effects on fungal diseases. Conversely, neomycin, kanamycin, and gentamicin primarily inhibit bacterial growth. This might explain the stronger resistance of af and jsh to black spot disease compared with qy.

### 3.3 Analysis of Jinggang honey pomelo transcriptome in response to *D. citri*

**Figure 4:**
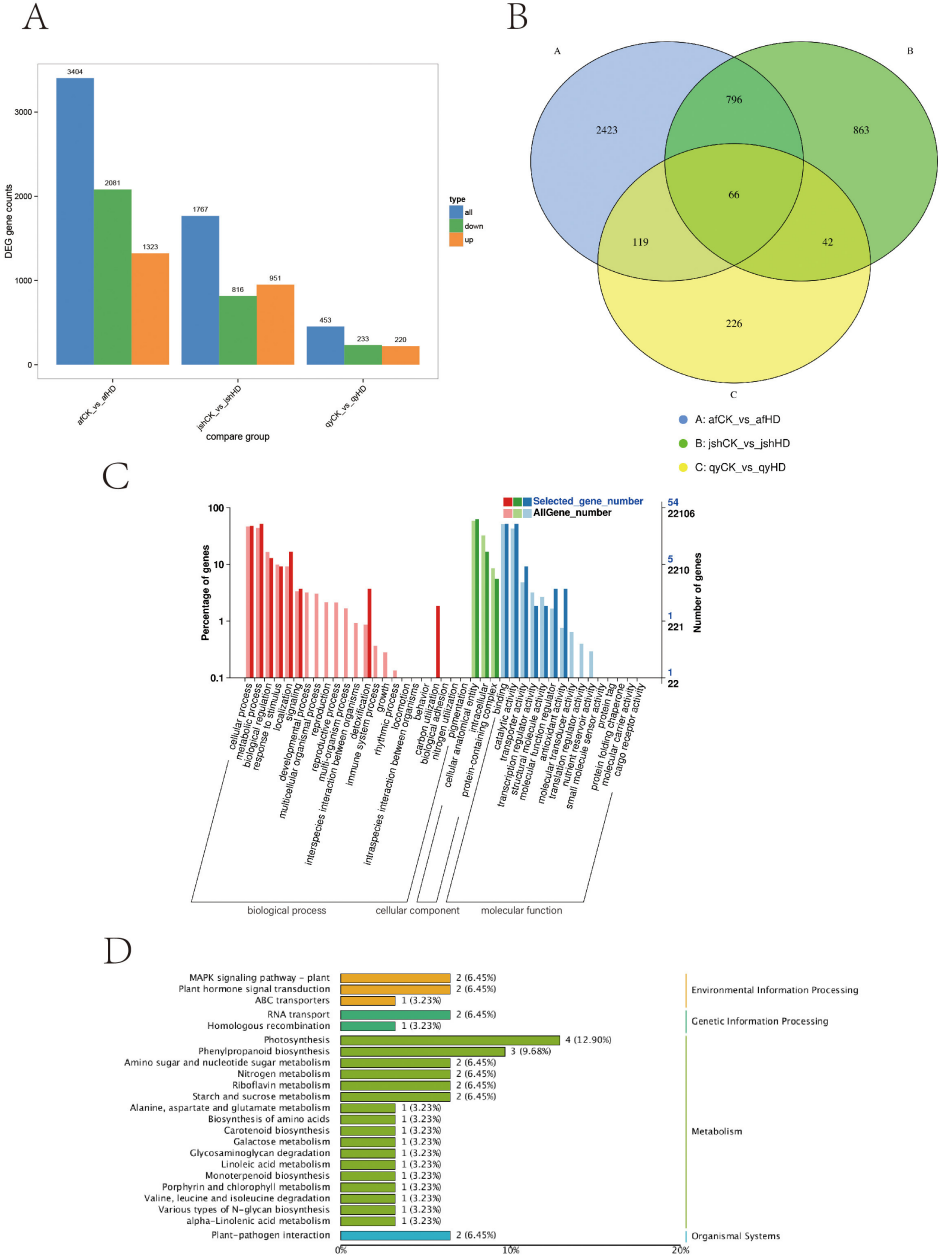
Analysis of Jinggang honey pomelo transcriptome in response to *D. citri*. **(A)** Number of DEGs between the three groups (afCK vs. afHD, jshCK vs. jshHD, and qyCK vs. qyHD); **(B)** Venn diagram showing DEGs shared among afCK vs. afHD, jshCK vs. jshHD, and qyCK vs. qyHD; **(C)** GO analysis of the 66 common DEGs; **(D)** KEGG analysis of the 66 common DEGs.

A total of 18 samples were prepared for transcriptome analysis, yielding a total of 133.88 Gb of clean data. Each sample's clean data amounted to 5.91 Gb, with guanine and cytosine content ranging from 43.81 to 44.60%, and Q30 ranging from 94.69 to 96.16%. More than 90.83% of the sequencing data in the sample aligned with the reference genome (Supplementary Table 2). A total of 3,404, 1,767, and 453 DEGs were identified in afCK vs. afHD, jshCK vs. jshHD, and qyCK vs. qyHD, respectively. Among these, 1,323, 951, and 220 DEGs were upregulated, whereas 2,081, 816, and 233 DEGs were downregulated (Figure 4A). To investigate the shared DEGs in response to black spot disease across different varieties of Jinggang honey pomelo, we analyzed the quantitative distribution of DEGs for afCK vs. afHD, jshCK vs. jshHD, and qyCK vs. qyHD. We identified a total of 66 DEGs across the three varieties (Figure 4B). Among the 66 common DEGs, 29 were upregulated in afCK vs. afHD, 31 in jshCK vs. jshHD, and 31 in qyCK vs. qyHD, while 37 were downregulated in afCK vs. afHD, 35 in jshCK vs. jshHD, and 35 in qyCK vs. qyHD (Supplementary Table 3).

The GO analysis of the 66 common DEGs revealed that these genes were predominantly enriched in cellular processes, cellular components, and binding functions, under the biological process (BP), cellular component (CC), and molecular function (MF) categories respectively (Figure 4C). The KEGG analysis of the 66 common DEGs showed these genes were significantly enriched in the photosynthesis and phenylpropanoid biosynthesis pathways (Figure 4D).

Photosynthesis plays a pivotal role in plant defense responses. As primary sources of reactive oxygen species (ROS) within plants, chloroplasts produce ROS such as hydrogen peroxide (H_2_O_2_) during photosynthesis. These ROS can act as signaling molecules to initiate defense responses against pathogenic invasion. Under biotic stress, plants typically down-regulate the expression of photosynthesis-related genes, which form part of the defense response (Hu et al., 2020).

In this study, the down-regulation of four photosynthesis-related common DEGs (Cg1g001670, Cg2g040770, Cg3g024680, and Cg4g017260) was observed in afCK vs. afHD, jshCK vs. jshHD, and qyCK vs. qyHD. This down-regulation could symbolize a trade-off between sustaining photosynthesis and triggering defense mechanisms in plants.

The phenylpropanoid biosynthesis pathway is a conduit for the synthesis of a variety of secondary metabolites in plants. The compounds produced by this pathway not only provide plants with physical and chemical protection against pathogenic invasion but also regulate defense signaling pathways (Yadav et al., 2020). In this study, the up-regulation of one common DEG associated with phenylpropanoid biosynthesis was observed in afCK vs. afHD, jshCK vs. jshHD, and qyCK vs. qyHD. This up-regulation might help plants to produce more secondary metabolites to counter biotic stress.

**Figure 5:**
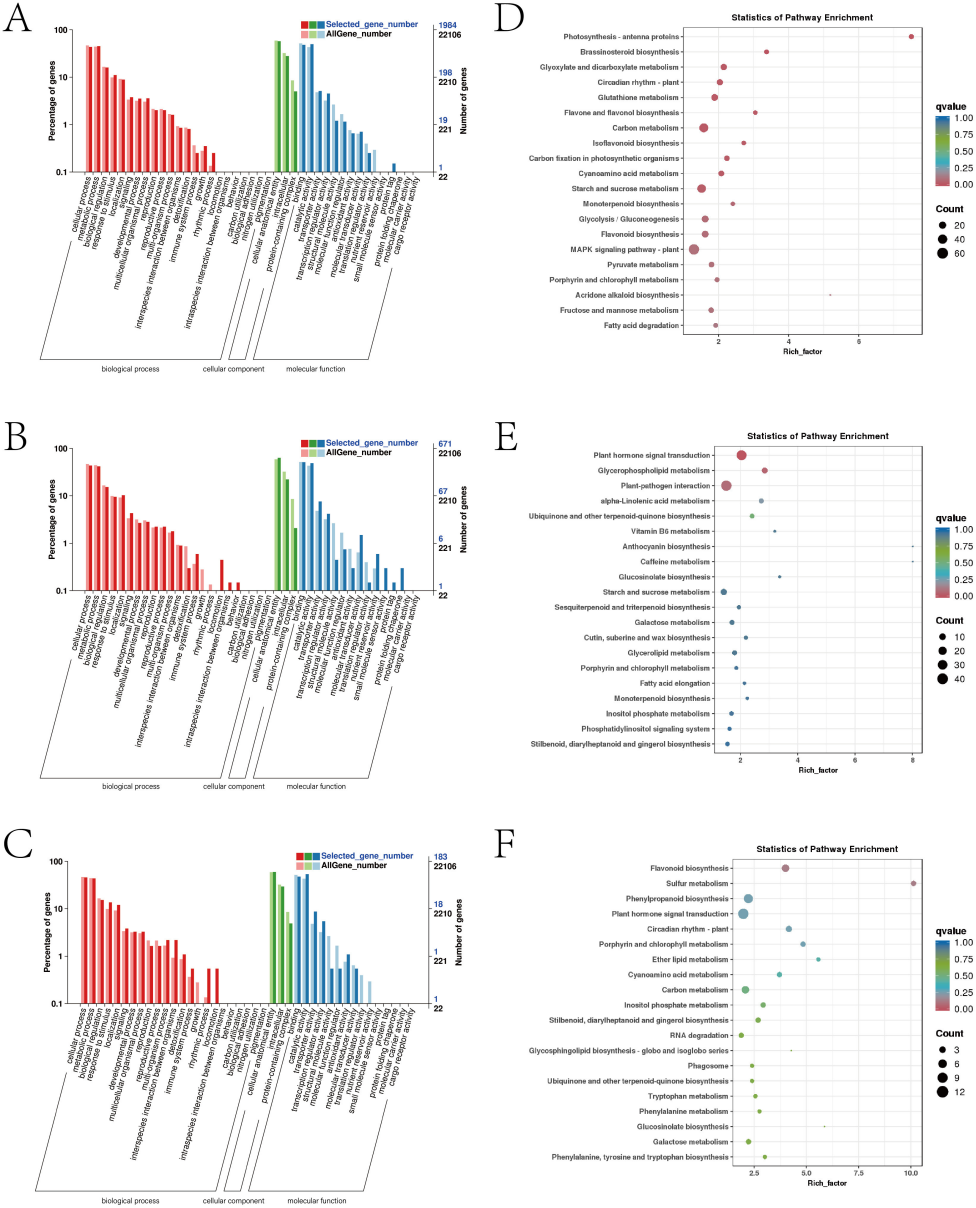
Analysis of Jinggang honey pomelo transcriptome in response to *D. citri*. **(A)** GO analysis of the specific DEGs in afCK vs. afHD; **(B)** GO analysis of the specific DEGs in jshCK vs. jshHD; **(C)** GO analysis of the specific DEGs in qyCK vs. qyHD; **(D)** KEGG analysis of the specific DEGs in afCK vs. afHD; **(E)** KEGG analysis of the specific DEGs in jshCK vs. jshHD; **(F)** KEGG analysis of the specific DEGs in qyCK vs. qyHD.

The GO analysis results of the DEGs exclusively present in the afCK vs. afHD, jshCK vs. jshHD, and qyCK vs. qyHD comparisons showed that in the BP category, these DEGs were primarily enriched in the cellular process, metabolic process, and biological regulation binding. In the CC category, the DEGs were mainly enriched in the cellular anatomical entity, intracellular, and protein-containing complex. In the MF category, these DEGs were predominantly enriched in binding, catalytic activity, and transporter activity (Figures 5A–C).

The results of the KEGG analysis demonstrated that two common pathways were enriched by the specific DEGs for afCK vs. afHD, jshCK vs. jshHD, and qyCK vs. qyHD: the photosynthesis and phenylpropanoid biosynthesis pathways. The DEGs were exclusive to afCK vs. afHD and were primarily enriched in the MAPK signaling pathway-plant and carbon metabolism. The DEGs exclusive to jshCK vs. jshHD were primarily enriched in plant hormone signal transduction and plant-pathogen interaction. Finally, the DEGs exclusive to qyCK vs. qyHD were primarily enriched in flavonoid biosynthesis and sulfur metabolism (Figures 5D–F).

### 3.4 Identification of differentially expressed TFs

TFs modulate gene expression by binding to functional gene regulatory regions, thus influencing various biological processes. TF prediction was carried out for 3,404, 1,767, and 453 DEGs in afCK vs. afHD, jshCK vs. jshHD, and qyCK vs. qyHD, identifying 214, 102, and 30 TFs, respectively. A total of 42, 29, and 5 DEGs in the *MYB* (25, 20, and 3 DEGs) and *bHLH* (17, 9, and 2 DEGs) TF families were detected, which are associated with flavonoid biosynthesis. Among these DEGs, 16 *MYBs* and three *bHLHs* exhibited high expression in afCK vs. afHD, 15 *MYBs* and six *bHLHs* in jshCK vs. jshHD, and two *MYBs* and one *bHLH* in qyCK vs qyHD (Supplementary Table 2).

### 3.5 DEGs and DAMs associated with the phenylpropanoid biosynthesis and flavonoid biosynthesis

**Figure 6:**
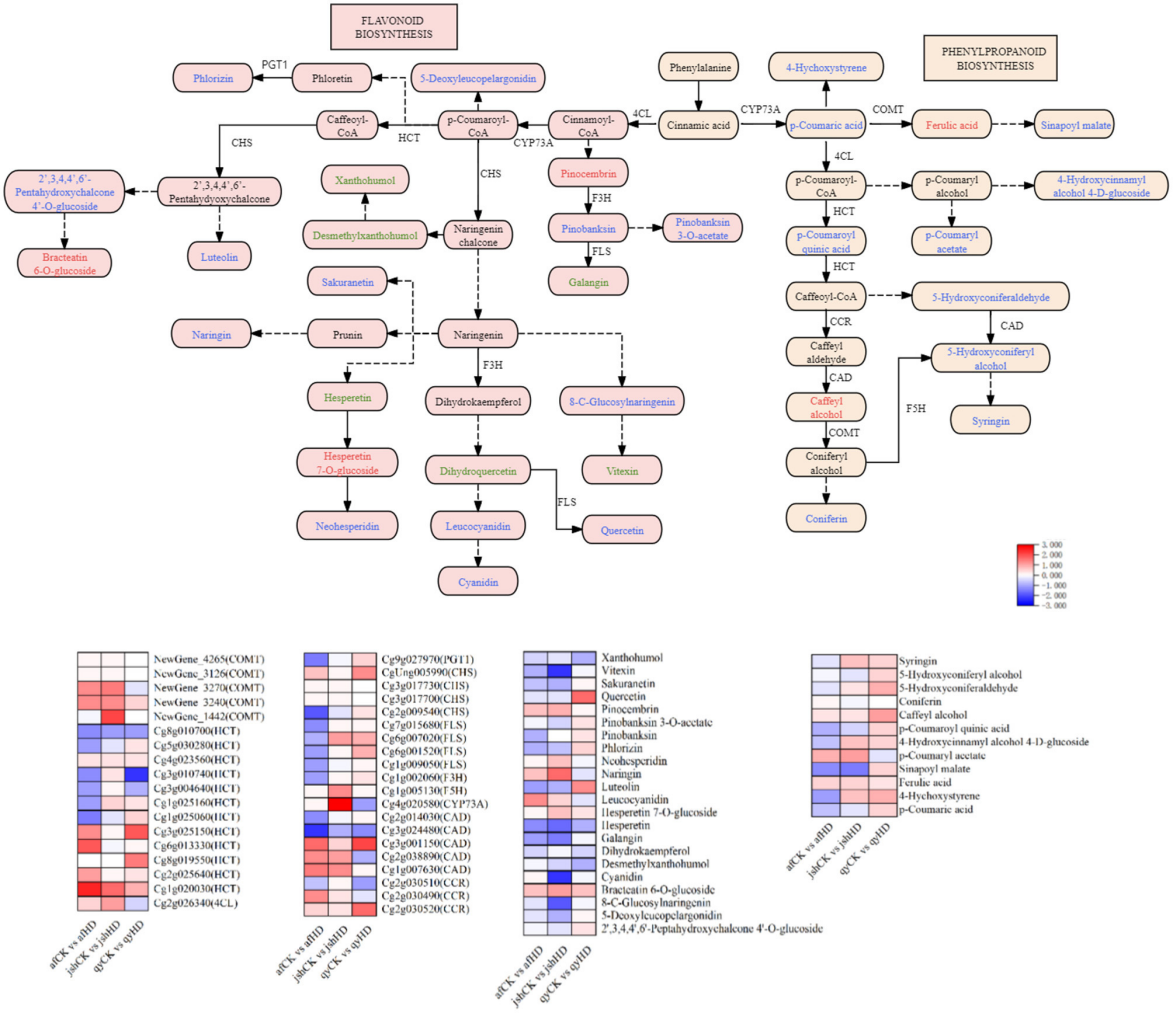
DEGs and DAMs associated with the phenylpropanoid biosynthesis and flavonoid biosynthesis; The red, green, and blue font indicates DAMs with up-regulated expression, DEGs with down-regulated expression, and DEGs with both up and down-regulated expression. Heatmap depicting DEGs and DAMs of phenylpropanoid biosynthesis and flavonoid biosynthesis.

To identify DEGs and DAMs associated with the synthesis of phytoalexin in the leaves of Jinggang honey pomelo infected with *D. citri*, we analyzed the phenylpropanoid and flavonoid biosynthesis pathways using combined metabolomic and transcriptomic data (Figure 6). Enrichment in the phenylpropanoid biosynthesis and flavonoid biosynthesis pathways was detected by 38 DEGs and 38 DAMs, specifically in the afCK vs. afHD, jshCK vs. jshHD, and qyCK vs. qyHD comparisons, post-infection with *D. citri*.

Among the 38 DEGs, two unigenes encoded caffeic acid 3-O-methyltransferase (COMT), four unigenes encoded shikimate O-hydroxycinnamoyltransferase (HCT), one unigene encoded 4-coumarate–CoA ligase (4CL), one unigene encoded ferulate-5-hydroxylase, one unigene encoded trans-cinnamate 4-monooxygenase (CYP73A), three unigenes encoded cinnamyl-alcohol dehydrogenase (CAD), and two unigenes encoded cinnamoyl-CoA reductase (CCR) in the afCK vs. afHD and jshCK vs. jshHD comparisons. All of these unigenes were upregulated. Correspondingly, the expression levels of ferulic acid, caffeyl alcohol, pinocembrin, p-coumaryl acetate, leucocyanidin, naringin, and neohesperidin also increased.

## 4 Discussion

*D. citri* is one of the most destructive fungal pathogens of citrus (Guarnaccia and Crous, 2017; Li-ying et al., 2012). It infects young leaves, shoots, and fruits, inducing black-to-reddish brown, raised pustules (known as melanose) on the leaves, twigs, and fruits of citrus (Nelson, 2008). While melanose typically does not decrease yield, it negatively affects the marketability of citrus fruits, leading to substantial economic losses (Li-ying et al., 2012; Rehman et al., 2020). Additionally, *D. citri* causes stem-end rot, shoot-blight, dieback, trunk or branch gummosis, and rot in all citrus species or varieties across the globe (Huang et al., 2013; Guarnaccia and Crous, 2017; Li-ying et al., 2012; Fawcett, 1912; Guo-Qing, 2010). Breeding disease-resistant varieties is often the most effective strategy for managing plant diseases. However, this approach requires an understanding of the interaction between Jinggang honey pomelo and *D. citri*, as well as the identification of disease-resistance genes in Jinggang honey pomelo. In this study, healthy and diseased fruits from three Jinggang honey pomelo lines - jsh, af, and qy, which exhibit different resistance levels to black spot disease, were analyzed using both transcriptomics and metabolomics. This combined analysis provides a unique opportunity to identify the candidate genes and metabolites involved in the disease-resistance pathway of Jinggang honey pomelo.

Previous studies suggest that physiological disorders can prompt significant changes in metabolite levels (Jung et al., 2015; Li et al., 2018). In the current study, 426 common DAMs and 66 collective DEGs were identified across three comparative groups and aligned to KEGG pathways. Key takeaways include the significantly enriched biosynthesis pathways of flavonoid compounds, namely phenylpropanoid biosynthesis and flavonoid biosynthesis. TFs are critical for the regulation of both growth and development in organisms, and play a part in responses to biotic and abiotic stress (Sun et al., 2022). Previous research has evidenced that *MYB* can mediate the transcription of key enzymes in the flavonoid synthesis pathway, thereby boosting flavonoid production (An et al., 2017). *bHLH* has been reported to have involvement in environmental stress responses and can regulate flavonoid biosynthesis in synergy with *MYB* (Liu et al., 2018; Wang et al., 2018). In the afCK vs. afHD and jshCK vs. jshHD groups, there were recorded 16 and 15 MYBs and three and six bHLHs, respectively, while only two MYBs and one bHLH were pinpointed in the qyCK vs. qyHD group. Drawn from the above examination, flavonoids, and their related biosynthetic pathways play a decisive role in the defense against *D. citri*.

The phenylpropane metabolic pathway starts from phenylalanine via the shikimic acid pathway. Phenylalanine is converted to p-coumaroyl-CoA by phenylalanine ammonia-lyase, cinnamic 4-hydroxylase (*C4H*), and 4-coumaroyl-CoA ligase (*4CL*), establishing a shared pathway for phenylpropane metabolism (Fraser and Chapple, 2011). Upon inoculation with *D. citri*, the expression of the *4CL* gene (Cg2g026340) was upregulated by 0.45 and 1.00 fold in the resistant varieties afCK vs. afHD and jshCK vs. jshHD, respectively. *4CL* catalyzes the conversion of p-coumaric acid into p-coumaroyl-CoA, a key step that introduces carbon atoms into the phenylpropanoid pathway, ultimately leading to the formation of various secondary metabolites (Wang et al., 2022). Subsequently, the reaction is catalyzed by other downstream enzymes, diverging into the two main branches of phenylpropane metabolism: the flavonoid pathway and the lignin pathway. The flavonoid pathway primarily synthesizes metabolites, including flavonoids, isoflavones, anthocyanins, and flavonols (Carletti et al., 2014). *CHS*, the first rate-limiting enzyme in the flavonoid biosynthesis pathway, catalyzes the condensation of p-coumaroyl-CoA with three molecules of malonyl-CoA to form chalcone. Subsequently, *CHI* catalyzes the isomerization of chalcone to flavanone, while *F3H* mediates the hydroxylation of flavanone at the C-3 position, yielding dihydroflavonol. Finally, *FLS* converts dihydroflavonol into flavonol, serving as the key enzyme for flavonol biosynthesis (Bulanov et al., 2025; Xie et al., 2023; Liu et al., 2021). Upon exposure to biological stress, plants primarily mitigate it by modulating the expression of genes related to the flavonoid pathway and accumulating various metabolites. However, the four unigenes (namely Cg2g009540, Cg3g017700, Cg3g017730, and CgUng005990) encoding CHS, one unigene (Cg1g002060) encoding *F3H*, and its encoded downstream products (including 2′, 3,4,4′, 6′-Pepperhydroxychalcone 4′ - O-glucoside, 5-Deoxyleucopelargonidin, Luteolin, Phlorizin, Pinobaksin, Pinobaksin 3-O-acetate, Quercetin, and Sakuranetin) were all upregulated in the susceptible varieties qyCK vs. qyHD compared with afCK vs. afHD and jshCK vs. jshHD. This may represent a compensatory mechanism by which plants attempt to enhance defense responses by increasing the synthesis of flavonoids (Ahmad et al., 2023).

The lignin biosynthesis pathway is a crucial metabolic route for plant cell wall formation, involving multiple key genes such as *HCT, C3H, CCR, CAD, F5H*, and *POD*. These genes play pivotal roles in lignin synthesis and regulation. Lignin is a major structural component of plant cell walls that enhances mechanical strength and forms a physical barrier against pathogen invasion. Upon pathogen infection, plants significantly increase lignin biosynthesis and deposition in a process termed lignification (Riseh et al., 2024). Previous phenotypic studies on af, jsh, and qy following *D. citri* infection revealed that lignification occurred specifically at the infection sites of af and jsh. The reinforcement of cell walls through lignification helps compartmentalize infected tissues, thereby restricting pathogen spread. In contrast, no lignification was observed in qy, where the infected areas displayed progressively expanding lesions over time after *D. citri* infestation. Additionally, numerous intermediates and related compounds in the lignin pathway exhibit notable antimicrobial activity. For instance, coumarins and stilbenes—synthesized via the lignin pathway—possess antibacterial and antioxidant properties that inhibit pathogen proliferation (Ma, 2024). Moreover, lignin monomers such as p-coumaric acid and ferulic acid can suppress fungal hyphal growth (Ninkuu et al., 2022). After infecting honey pomelos with *D. citri*, the four unigenes (Cg1g020030, Cg2g025640, Cg8g019550, and Cg3g025150) encoding *HCT*, two unigenes (Cg2g030520 and Cg2g030490) encoding *CCR*, three unigenes (Cg1g007630, Cg2g038890, and Cg2g030490) encoding *CAD*, and one unigene (Cg1g005130) encoding *F5H*, along with the levels of lignin precursors (p-coumaryl acetate, pinocembrin, naringin, and neohesperidin) were all upregulated in afCK vs. afHD and jshCK vs. jshHD. These findings suggest that plants may enhance pathogen resistance by upregulating key genes and metabolites in the lignin pathway, thus strengthening cell wall physical defenses, activating signaling pathways, and maintaining metabolic accumulation with feedback regulation.

## 5 Conclusions

In summary, the transcriptome and metabolome of fruit epidermis infected by *D. citri* at 13 days post-infection (dpi) were profiled using RNA-seq and UPLC-MS/MS, respectively. Comparisons between afCK and afHD, jshCK and jshHD, and qyCK and qyHD exhibited higher levels of DEGs and DAMs post-infection, showing 1,744, 1,616, and 1,325 DAMs, and 3,403, 1,767, and 453 DEGs, respectively. This indicates that their defense responses were activated. These DEGs and DAMs were then mapped to KEGG pathways, including phenylpropanoid and flavonoid biosynthesis. Additionally, *MYB* and *bHLH* TFs connected with these pathways were highly expressed. Furthermore, the genes and metabolites in this pathway could play a crucial role in the resistance of Jinggang honey pomelo to the black spot pathogen (*D. citri*). However, the disease resistance of Jinggang honey pomelo involves a complex series of regulatory and signaling mechanisms. Overall, this study sheds light on the defense mechanisms of Jinggang honey pomelo against black spot disease, contributing to the detection of molecular markers for resistance and aiding in the cultivation of Jinggang honey pomelo varieties with enhanced black spot disease resistance.

## Data Availability

The raw data supporting the conclusions of this article will be made available by the authors, without undue reservation.
