# Supplementary material for: An integrated transcriptomic and metabolomic analysis of black spot disease in Jinggang honey pomelo reveals underlying resistance mechanisms
Source: Front Microbiol. 2025 May 15;16:1495804. doi: 10.3389/fmicb.2025.1495804 (PMC12119630; doi:10.3389/fmicb.2025.1495804)
Supplement: Supplementary file 1 [file Data_Sheet_1.docx]

Supplementary Material


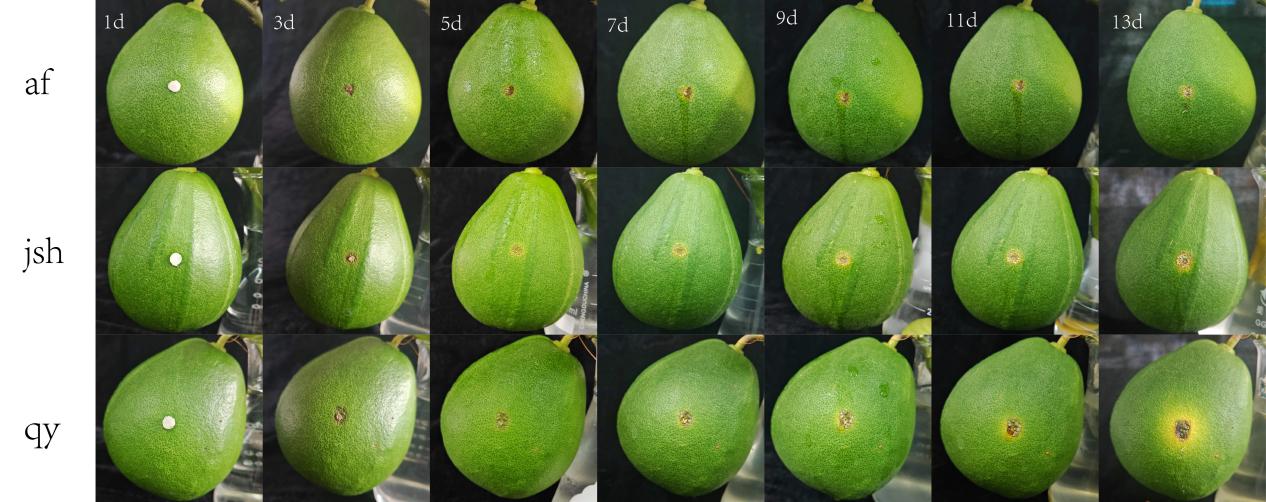


**Figure S1**. Phenotype of af, jsh, and qy at 1, 3, 5, 7, 9, 11, and 13 days after inoculation with *D.citri*

**Table S1.** Regulation of 426 common DAMs in afCK vs. afHD, jshCK vs. jshHD, and qyCK vs. qyHD

| #ID | name | afCK vs afHD | jshCK vs jshHD | qyCK vs qyHD |
| --- | --- | --- | --- | --- |
| neg_1089 | Bavachin | down | down | down |
| neg_1155 | Sibiricose A3 | down | down | up |
| neg_1161 | Strictosidine aglycone | down | down | down |
| neg_1175 | 5-Hydroxy-N-formylkynurenine | up | up | up |
| neg_1203 | 1-Salicylate glucuronide | up | down | down |
| neg_1205 | Hetacillin | up | up | up |
| neg_1213 | cis-trans-Nepetalactol | up | up | up |
| neg_1214 | S-Methyl-L-cysteine | up | up | up |
| neg_1240 | 4,8-Dihydroxyquinoline | up | up | up |
| neg_1293 | Cys-Gly | up | up | up |
| neg_1324 | Strigol | down | down | down |
| neg_1373 | 3,4,5-Tricaffeoylquinic acid | up | up | up |
| neg_1378 | Dimetghyl 4-Hydroxyisophthalate | down | down | up |
| neg_1434 | Chrysoeriol-6-C-glucoside | down | down | up |
| neg_1436 | Gamma-Mangostin | down | down | down |
| neg_1438 | Licoisoflavone A | down | down | down |
| neg_1470 | 6-Oxo-1,4,5,6-tetrahydronicotinate | up | up | up |
| neg_1474 | Roquefortine C | up | up | up |
| neg_1485 | cis-3-(Carboxy-ethyl)-3,5-cyclo-hexadiene-1,2-diol | down | up | down |
| neg_1546 | Geranyl diphosphate | up | down | down |
| neg_1556 | scopoletin-7-O-glucoside (Scopolin) | down | down | down |
| neg_1559 | 4-(3-Methylbut-2-enyl)-L-tryptophan | up | up | down |
| neg_1573 | 6-(Hydroxymethyl)-7,8-dihydropterin | up | up | down |
| neg_1578 | Dihydrolipoyl-AMP | up | up | down |
| neg_1583 | (R)-3-((R)-3-Hydroxybutanoyloxy)butanoate | down | up | up |
| neg_1603 | Feruloylagmatine | up | up | up |
| neg_1609 | Vaccarin | down | down | up |
| neg_1628 | Dihydrokaempferol-3-O-glucoside | up | up | down |
| neg_1649 | L-Arabinono-1,4-lactone | down | down | down |
| neg_1675 | Quercetin-3-O-methyl ether | down | down | up |
| neg_1678 | Robinin | up | up | down |
| neg_1702 | Deoxyguanidinoproclavaminic acid | down | down | up |
| neg_1704 | Harpagide | up | up | up |
| neg_1753 | Malonylapiin | down | down | up |
| neg_1774 | Prunasin | up | up | up |
| neg_1795 | Pinnatifinoside A | up | down | up |
| neg_1807 | Daidzein-7-O-apiosyl(1?6)glucoside | down | down | up |
| neg_1823 | Tripterygiumine M | up | up | up |
| neg_1835 | Icariside B1 | down | down | down |
| neg_1888 | Apigenin-7-O-(6''-acetyl)glucoside | down | down | up |
| neg_1930 | Sinapoyl malate | down | down | up |
| neg_1946 | Dihydrodemethylsterigmatocystin | down | down | up |
| neg_1957 | 1'-Hydroxyversicolorone | down | down | up |
| neg_1965 | Dopaxanthin quinone | down | down | up |
| neg_2031 | S-Nitroso-L-glutathione | down | down | up |
| neg_2069 | Nortrachelogenin-4-O-glucoside | up | up | up |
| neg_2082 | 4,5-Dicaffeoylquinic acid | down | down | up |
| neg_2163 | 2-(4-Aminobutanamido)-3-(1-methyl-1H-imidazol-5-yl)propanoic acid | up | up | down |
| neg_2176 | Maduropeptin chromophore | down | down | down |
| neg_2188 | Mulberroside A | down | up | down |
| neg_2204 | 2,3-Dihydrothienamycin | down | down | down |
| neg_2236 | 5,7,4'-Trihydroxy-3,6,3',5'-Tetramethoxyflavone | down | down | up |
| neg_2249 | 7-Aminomethyl-7-carbaguanine | up | up | down |
| neg_2255 | Meliarachin B | up | up | up |
| neg_2294 | Radicamine B | down | up | down |
| neg_2298 | 3,6'-Diferuloylsucrose | up | up | down |
| neg_2350 | Lumazine | down | down | up |
| neg_2356 | Oxypaeoniflorin | down | down | down |
| neg_2399 | Kaempferol 3-O-[6-(4-coumaroyl)-beta-D-glucosyl-(1->2)-beta-D-glucosyl-(1->2)-beta-D-glucoside] | up | down | up |
| neg_2428 | Bilobalide | up | up | up |
| neg_2432 | Luteolin-7-O-neohesperidoside (lonicerin) | down | down | down |
| neg_2464 | Roseoside | up | down | down |
| neg_2485 | Daidzin | up | up | up |
| neg_2503 | Jasmonic acid | up | up | up |
| neg_2600 | HAEMATOMMIC ACID, ETHYL ESTER | up | up | up |
| neg_2611 | Quercetin-3-O-gentiotetroside | down | down | up |
| neg_2613 | 4-(2-Aminophenyl)-2,4-dioxobutanoate | down | down | up |
| neg_2615 | Oroxylin A | down | down | up |
| neg_2620 | 6''-O-Malonylgenistin | down | down | up |
| neg_2624 | 183927 | down | down | up |
| neg_2637 | Kaempferol-3-O-sambubioside | down | down | up |
| neg_2796 | Yersiniabactin | up | up | up |
| neg_2838 | PSOROMIC ACID | up | down | down |
| neg_2853 | N-Acetylaspartylglutamylglutamate | up | up | up |
| neg_2877 | Naringenin-4',7-dimethyl ether | down | down | down |
| neg_2884 | Magnesium protoporphyrin | up | up | down |
| neg_2972 | Rubitecan | up | up | up |
| neg_3089 | Phthalate 3,4-cis-dihydrodiol | down | up | down |
| neg_3153 | 2-Acetamido-4-(D-alanylamino)-2,4,6-trideoxy-D-mannopyranose | up | up | down |
| neg_3172 | 3,5-Dicaffeoylquinic acid | up | up | up |
| neg_3176 | Estrone 3-glucuronide | down | up | down |
| neg_3191 | Premithramycinone | down | up | down |
| neg_3264 | Astilbin | up | up | up |
| neg_3412 | Flavanomarein | down | down | up |
| neg_3420 | Isoacteoside | down | up | up |
| neg_3422 | Protodeoxyviolaceinic acid | down | up | up |
| neg_3442 | 6'-(2,3-Dihydroxybenzoyl)sweroside | down | down | up |
| neg_3479 | Diosmin | down | down | up |
| neg_3483 | Bracteatin 6-O-glucoside | up | up | up |
| neg_3493 | Premithramycin A3' | down | down | up |
| neg_3614 | ageconyflavone B | down | down | up |
| neg_3629 | Mudanoside A | down | down | down |
| neg_3636 | Hesperetin | down | down | down |
| neg_3640 | 5-Methylthio-D-ribose | down | down | up |
| neg_3648 | Eriodictyol-7-O-glucoronide | up | up | up |
| neg_373 | Metronidazole | up | up | down |
| neg_3799 | Olmesartan | up | up | up |
| neg_3865 | Sparfloxacin | up | up | up |
| neg_3878 | Angelicide | up | up | down |
| neg_3890 | Centaurein | up | up | up |
| neg_3913 | Astemizole | up | up | up |
| neg_3919 | TRIBOA-glucoside | down | down | up |
| neg_3925 | Norsolorinic acid anthrone | down | down | up |
| neg_3938 | Isolariciresinol-9'-O-rhamnoside (Aviculin) | up | down | up |
| neg_3942 | Arginyl-Aspartate | up | down | down |
| neg_4029 | 2,3-Dinor-8-iso prostaglandin F1alpha | up | up | up |
| neg_4035 | Isorhoifolin | down | down | up |
| neg_4048 | N-Demethyl-desepoxymaytansinol | down | down | up |
| neg_4051 | Myristoyl-coenzyme A | down | down | up |
| neg_4093 | 7-Deoxyloganic acid | down | down | up |
| neg_4098 | Acadesine (Drug) | up | down | up |
| neg_4126 | Rhapontin | down | down | up |
| neg_4143 | 4-Methoxy-2,2'-bipyrrole-5-carbaldehyde | up | up | up |
| neg_4145 | 1,3-Dihydroxy-N-methylacridone | up | up | up |
| neg_4155 | 9,10-Dihydroxy-12,13-epoxyoctadecanoate | up | up | up |
| neg_4211 | Gibberellin A4 | up | up | down |
| neg_4212 | (1R,6S)-6-Amino-5-oxocyclohex-2-ene-1-carboxylate | up | up | down |
| neg_4250 | 17-beta-Estradiol-3-glucuronide | up | down | up |
| neg_4252 | Ssioriside | up | down | up |
| neg_4256 | 2-Hydroxyadenosine | up | down | up |
| neg_4286 | 8-Demethyl-8-(methylamino)riboflavin | up | up | up |
| neg_4290 | 17-(Furan-3-yl)-7-hydroxy-4,4,8-trimethylandrosta-1,14-diene-3,16-dione (Nimbocinol) | up | down | up |
| neg_4315 | Pterostilbene | up | up | up |
| neg_4344 | Hesperetin-7-O-neohesperidoside(Neohesperidin) | up | up | up |
| neg_4424 | (-)-Jasmonoyl-L-isoleucine | up | up | up |
| neg_4475 | Nicotinamide-beta-riboside | up | up | up |
| neg_448 | Lipoic acid | up | up | down |
| neg_4488 | L-Olivosyl-oleandolide | up | down | up |
| neg_4556 | Chikusetsusaponin IVa | up | down | down |
| neg_4561 | Hydrogenobyrinate a,c diamide | up | down | down |
| neg_4566 | Senkyunolide R | up | up | up |
| neg_4640 | Streptomycin | up | up | up |
| neg_4692 | Ractopamine | up | up | up |
| neg_4698 | Ergotamine | up | up | up |
| neg_4728 | 9(S)-HOTrE | up | up | up |
| neg_4804 | Dobutamine | down | up | up |
| neg_4807 | N6-(2-Hydroxyethyl)adenosine | down | down | down |
| neg_4817 | gamma-L-Glutamyl-L-2-aminobutyrate | up | up | up |
| neg_4818 | Isoleucyl-Tryptophan | up | up | up |
| neg_4820 | 7'-O-Dmethylcephaeline | down | down | up |
| neg_4821 | 10-OPDA | up | up | up |
| neg_4917 | (2R,3R)-3-Methylglutamyl-5-semialdehyde-N6-lysine | up | down | down |
| neg_4949 | Bilirubin beta-diglucuronide | down | down | down |
| neg_4963 | 6'''-Deamino-6'''-oxoneomycin C | up | up | up |
| neg_4975 | bryophyllin B | up | up | up |
| neg_5016 | Cucurbitacin B | up | up | up |
| neg_537 | 2-O-Salicyl-6-O-Galloyl-D-Glucose | down | down | down |
| neg_723 | 3-Hydroxy-4H-pyran-4-one | up | up | up |
| neg_742 | L-Adrenaline | down | down | up |
| neg_765 | choerospondin | down | down | down |
| neg_807 | N-((R)-Pantothenoyl)-L-cysteine | up | up | up |
| pos_1705 | 1-O-Galloyl-D-glucose | up | down | up |
| pos_1720 | O-Methylandrocymbine | down | down | down |
| pos_1805 | Dehydrocostuslactone | up | up | up |
| pos_1813 | safflor yellow A | down | down | up |
| pos_1840 | N-Methylhydantoin | up | up | up |
| pos_1841 | 2-Naphthylamine | up | up | up |
| pos_1842 | Adipic acid | up | up | up |
| pos_1843 | Indoleacetaldehyde | up | up | up |
| pos_1845 | 3-amino-2-naphthoic acid | up | up | up |
| pos_1858 | Versiconol | down | down | up |
| pos_1900 | Sinapine | down | down | up |
| pos_1908 | Tyrosyl-Phenylalanine | up | up | up |
| pos_1941 | Aurantio-obtusin-6-O-Glucoside | down | down | down |
| pos_1994 | 5-Phenyl-1,3-oxazinane-2,4-dione | up | up | up |
| pos_2026 | Imidazole lactate | up | up | up |
| pos_2032 | 3-Formyl-6-hydroxyindole | up | up | up |
| pos_2042 | (1R*,2R*,4R*,8S*)-p-Menthane-1,2,8,9-tetrol 9-glucoside | up | up | up |
| pos_2054 | UDP-2-acetamido-2-deoxy-alpha-D-ribo-hex-3-uluronate | down | down | down |
| pos_2062 | 4,21-Dehydrogeissoschizine | up | up | up |
| pos_2065 | Perseitol | up | up | up |
| pos_2087 | trans-Caffeic acid [apiosyl-(1->6)-glucosyl] ester | down | down | up |
| pos_2090 | dTDP-L-megosamine | down | down | up |
| pos_2101 | L-Rhamnono-1,4-lactone | down | down | down |
| pos_2103 | Homatropine | down | down | down |
| pos_2112 | Kinetin | up | down | up |
| pos_2114 | Taxifolin-3'-O-glucoside | down | down | up |
| pos_2149 | Aminopterin | down | down | down |
| pos_2154 | 2-Methoxy-4-methylphenol | down | down | up |
| pos_2159 | 7-Methoxy-5,6:3',4'-bis(methylenedioxy)flavone | down | down | up |
| pos_2161 | Meliternatin | down | down | up |
| pos_2166 | BL V | down | down | up |
| pos_2170 | L-2-Aminoadipate adenylate | down | down | up |
| pos_2171 | Betanin | down | down | up |
| pos_2174 | Luteolin 6-C-glucoside 8-C-arabinoside | down | down | up |
| pos_2178 | Quercetin 3-O-[beta-D-xylosyl-(1->2)-beta-D-glucoside] | down | down | up |
| pos_2187 | Farrerol-7-O-glucoside | up | up | up |
| pos_2192 | 3-(5'-Methylthio)pentylmalic acid | up | up | up |
| pos_2194 | Aspartame | up | up | up |
| pos_2201 | Allamandin | down | down | up |
| pos_2205 | Ampelopsin A | down | down | up |
| pos_2222 | 4-Hydroxybenzyl alcohol | down | down | up |
| pos_2223 | 3-Acetyldihydro-2(3H)-furanone | down | down | up |
| pos_2227 | Mahaleboside | down | down | up |
| pos_2238 | Momilactone A | down | down | down |
| pos_2253 | Tryptophol | up | up | up |
| pos_2256 | 3,4,5,2',4',6'-Hexahydroxychalcone 2'-glucoside | down | down | up |
| pos_226 | (N-Sulfoindol-3-yl)methylglucosinolate | down | down | down |
| pos_2285 | Levanbiose | up | down | down |
| pos_2287 | N3-Fumaroyl-L-2,3-diaminopropanoate | down | down | down |
| pos_2299 | domesticoside | down | down | up |
| pos_2304 | Dictangustine A | up | up | up |
| pos_2310 | Derhamnosylmaysin | down | down | up |
| pos_2319 | Kaempferol 3,5-diglucoside | down | down | up |
| pos_2321 | Quercetin 3-sambubioside-7-rhamnoside | down | down | up |
| pos_2324 | L-Glutamate | up | down | down |
| pos_2325 | Hydroquinine | up | down | up |
| pos_2341 | Homovanillyl alcohol | up | up | up |
| pos_2343 | Palmitoleic Acid | down | down | down |
| pos_2351 | Ferulamide | up | up | up |
| pos_2354 | 3',4'-Methylenedioxy-alpha-pyrrolidinopropiophenone | up | up | up |
| pos_2379 | O-Methylsterigmatocystin | down | down | up |
| pos_2381 | methyl chlorogenate | down | down | up |
| pos_2391 | Uridine | down | down | up |
| pos_2425 | petunidin-3-O-arabinoside | down | down | up |
| pos_2474 | (8R,8'R,9S)-9-Hydroxy-3,4-dimethoxy-3',4'-methylenoxy-9,9'-epoxylignan | up | up | up |
| pos_2475 | Benzyl primeveroside | down | down | up |
| pos_2482 | Nortrachelogenin | up | up | up |
| pos_2492 | Atropaldehyde | down | down | up |
| pos_2543 | 11-O-Demethyl-17-O-deacetylvindoline | down | down | down |
| pos_2549 | (3R)-3-Hydroxy-2,3-dihydrotabersonine | up | up | up |
| pos_2572 | Kaempferol-3-O-(6''-Rhamnosyl-2''-Glucosyl)Glucoside (Camelliaside A) | down | down | up |
| pos_2576 | Securinine | up | up | up |
| pos_2582 | 5'-Phosphoribosylglycinamide | down | down | up |
| pos_2608 | Urate-3-ribonucleoside | down | down | up |
| pos_265 | 3-[(1R,2S,5R,6S)-5-Hydroxy-7-oxabicyclo[4.1.0]heptan-2-yl]-2-oxopropanoate | up | up | down |
| pos_2680 | Quercetin 3,7-dirhamnoside | down | down | up |
| pos_2691 | Ginkgolide J | up | up | up |
| pos_2694 | 2,2-Bis(4-hydroxyphenyl)hexafluoropropane | down | down | up |
| pos_2695 | 5-Amino-6-(5'-phospho-D-ribitylamino)uracil | down | down | up |
| pos_2703 | CMP-3-deoxy-D-manno-octulosonate | down | down | up |
| pos_2731 | Neobanol | down | down | up |
| pos_2732 | Dalspinin | down | down | up |
| pos_2733 | Daphnoretin | down | down | up |
| pos_2734 | Nogalonate | down | down | up |
| pos_2735 | Tetracenomycin B3 | down | down | up |
| pos_2736 | Kaempferol 3-O-glucoside | down | down | up |
| pos_2742 | Phosphatidylserine | up | up | down |
| pos_2757 | Acetyl vitamin K5 | up | up | up |
| pos_276 | Phenylalanyl-Glutamine | up | down | up |
| pos_2764 | Phellamurin | down | down | down |
| pos_2769 | O-Demethyl-N-demethyl-staurosporine | down | down | down |
| pos_2783 | Urdamycinone B | down | down | up |
| pos_2785 | Luteolinidin chloride | down | down | up |
| pos_2804 | 3-Methoxy-4,5-methylenedioxycinnamaldehyde | up | up | up |
| pos_2853 | 6-C-Galactosylisoscutellarein | down | down | up |
| pos_2905 | Biondnoid I | down | down | up |
| pos_2920 | Citrusin B | up | up | down |
| pos_2937 | Asp Thr Phe Trp | up | up | up |
| pos_2959 | cannflavin A | up | up | up |
| pos_2963 | beta-Rhodomycin | down | down | up |
| pos_2982 | Vincristine | up | up | up |
| pos_2986 | 4-caffeoylshikimic acid | down | down | up |
| pos_3026 | Dopamine quinone | down | down | down |
| pos_3045 | Mitomycin | up | down | down |
| pos_3053 | Hydroxypyruvic acid | up | down | down |
| pos_3061 | Adriamycinone | down | down | up |
| pos_3067 | Kaempferol 7,4'-dirhamnoside | down | down | up |
| pos_3086 | Irisolone | down | down | up |
| pos_3090 | Isovitexin | down | down | up |
| pos_3110 | Glu Phe Met Phe | up | up | up |
| pos_3157 | C-1027 Chromophore | down | up | down |
| pos_3193 | Dalpatein | up | up | up |
| pos_3204 | p-coumaric acid methyl ester | down | down | up |
| pos_3207 | Apigenin 7-O-beta-D-glucoside | down | down | up |
| pos_3224 | 3,7,4'-Tri-O-methylquercetin | down | down | up |
| pos_3226 | 3-Hydroxy-2-methylpyridine-5-carboxylate | down | down | up |
| pos_3234 | Dalspinin 7-O-galactoside | down | down | up |
| pos_3251 | Isookaninrhamnoside | down | down | up |
| pos_3267 | 4''-O-Carbamoyl ansamitocinoside P-3 | up | up | up |
| pos_3268 | Hydrangeifolin I | up | up | up |
| pos_3281 | N-Acetyl-D-glucosamine 6-phosphate | up | up | up |
| pos_3284 | S-Glutathionyl-L-cysteine | up | up | up |
| pos_3304 | Gly-Asn | down | down | up |
| pos_3309 | N-Succinyl-L-glutamate | down | down | up |
| pos_3330 | Ser Gly Trp Tyr | down | down | down |
| pos_3347 | Indolelactic acid | down | down | up |
| pos_3377 | (E)-m-Coumaric acid | down | up | up |
| pos_3390 | 100-1 | up | up | up |
| pos_3393 | Amaranthin | down | down | up |
| pos_3421 | O-(1->4)-alpha-L-Dihydrostreptosyl-streptidine 6-phosphate | down | down | up |
| pos_3438 | Penduletin (5,4'-Dihydroxy-3,6,7-trimethoxyflavone) | up | up | up |
| pos_347 | D-Quinovose | up | up | down |
| pos_3485 | N-Acetylglucosaminylasparagine | up | down | down |
| pos_3519 | Isoligustroside | up | down | down |
| pos_3579 | Pelargonidin 3-O-(6-O-malonyl-D-glucoside) | down | down | up |
| pos_3599 | 1-O-Caffeoyl-3-O-p-coumaroylglycerol | up | up | up |
| pos_3602 | 4-O-Galloylarbutin | up | up | down |
| pos_3651 | D-Lactose | up | down | up |
| pos_3736 | Methylenedioxycinnamic acid | up | up | up |
| pos_3781 | Meleagrin | down | down | down |
| pos_3790 | Cirsiliol (3',4',5-Trihydroxy-6,7-Dimethoxyflavone) | down | down | up |
| pos_3814 | Gentianamine | down | down | up |
| pos_3829 | Molephantin | down | down | up |
| pos_3841 | Malvidin-3-(p-coumaroyl)-rutinoside-5-glucoside | up | up | up |
| pos_3922 | Proline betaine | down | down | up |
| pos_3929 | Kadsuralignan H | down | down | up |
| pos_3965 | gamma-L-Glutamyl-L-propargylglycine | up | up | up |
| pos_3993 | F-Honaucin A | down | down | up |
| pos_4073 | Wilfordinine G | up | up | up |
| pos_4183 | Evonine | down | down | up |
| pos_4199 | Genipin-1-O-gentiobioside | up | up | up |
| pos_4242 | 1-Methylpiperidine-2-carboxylic acid | down | down | up |
| pos_4247 | N2-Succinyl-L-ornithine | up | up | up |
| pos_4259 | Quercetin-3-O-neohesperidoside | down | down | up |
| pos_4281 | (E)-4-Hydroxyphenylacetaldehyde oxime | up | up | up |
| pos_4291 | Petunidin 3-O-glucoside | up | down | up |
| pos_4306 | 4',5,7,8-TETRAHYDROXYFLAVANONE | down | down | up |
| pos_4311 | Kaempferol 3-O-beta-D-glucosyl-(1->2)-beta-D-glucoside | down | down | up |
| pos_4316 | Dunalianoside A | up | down | up |
| pos_4345 | Canthoside A | up | up | up |
| pos_4374 | 6'-O-Feruloyl-D-sucrose | up | up | up |
| pos_442 | N1-Methyl-4-pyridone-5-carboxamide | up | up | down |
| pos_4444 | Hydroxymethylbilane | down | down | up |
| pos_4445 | Urdamycin F | up | up | up |
| pos_445 | N6-Acetyl-L-lysine | up | up | up |
| pos_4451 | cis-3-(3-Carboxyethenyl)-3,5-cyclohexadiene-1,2-diol | down | down | up |
| pos_450 | Valienone | up | down | down |
| pos_4520 | Eugenin | up | up | up |
| pos_456 | N-Acetylmuramate | up | up | down |
| pos_4584 | 1-O-Galloyl-2-O-p-Coumaroyl-D-glucose | up | up | up |
| pos_4585 | N6,N6,O-Tridemethylpuromycin-5'-phosphate | down | up | up |
| pos_4620 | Kaempferol 3-sophorotrioside | up | up | up |
| pos_4629 | cis-3,4-Leucopelargonidin | down | down | down |
| pos_4638 | Chrysin 7-rutinoside | down | down | down |
| pos_4654 | wilfortrine | down | down | up |
| pos_4656 | Baccatin III | up | up | up |
| pos_468 | (R)-(+)-2-Pyrrolidone-5-carboxylic acid | up | up | down |
| pos_4725 | Arginyl-Tyrosine | up | up | up |
| pos_4732 | 3-Methylthiopropyl-desulfoglucosinolate | down | down | up |
| pos_4745 | UDP-3-O-(3-hydroxytetradecanoyl)-N-acetylglucosamine | down | down | up |
| pos_4751 | Pinocembrin-7-O-glucoside | up | up | up |
| pos_4762 | S-Formylglutathione | down | down | up |
| pos_4764 | Delphinidin-3-O-rutinoside-7-O-glucoside | up | up | up |
| pos_4778 | Gibberellin A19 | up | down | down |
| pos_4832 | Cinnamoylglycine | up | up | up |
| pos_4843 | Demethyldecarbamoylnovobiocin | down | up | up |
| pos_4860 | L-1-Pyrroline-3-hydroxy-5-carboxylate | down | down | down |
| pos_4885 | Violacein | up | down | down |
| pos_4898 | Sesaminol 2-O-Beta-D-Glucoside | up | up | up |
| pos_4909 | Cyanidin 5-O-beta-D-glucoside 3-O-beta-D-sambubioside | up | up | up |
| pos_4977 | Myricetin-3-O-galactoside | down | up | down |
| pos_5000 | 3,6-Dihydronicotinic acid | down | down | up |
| pos_5034 | Syringin | down | up | up |
| pos_5065 | Bisnorbiotin | down | down | up |
| pos_5070 | 1-O-Glucosyl sinapate | down | down | up |
| pos_5075 | Roquefortine D | up | up | down |
| pos_508 | (2E,6Z,9Z,12Z,15Z,18Z,21Z)-Tetracosaheptaenoyl-CoA | up | up | down |
| pos_513 | AminoDHQ | up | up | down |
| pos_521 | Ile-Ala-Arg | up | up | up |
| pos_5238 | Deoxytubulosine | down | down | up |
| pos_5275 | N2-Citryl-N6-acetyl-N6-hydroxy-L-lysine | down | down | up |
| pos_5363 | (E)-4-(Trimethylammonio)but-2-enoate | down | down | up |
| pos_5414 | Benomyl | down | down | up |
| pos_5416 | Reduced riboflavin | down | down | up |
| pos_545 | Acefylline; Theophylline-7-acetic acid | up | down | down |
| pos_5453 | 8-Oxodiacetoxyscirpenol | down | down | up |
| pos_5455 | C20817 | down | down | up |
| pos_5458 | Naringenin-7-O-Rutinoside(Narirutin) | down | down | up |
| pos_5495 | Glicoisoflavanone | down | down | down |
| pos_551 | DL-Propargylglycine | up | down | down |
| pos_5530 | dTDP-D-forosamine | down | down | up |
| pos_5541 | S-Adenosylmethionine | down | down | up |
| pos_5544 | Methionyl-Arginine | up | up | up |
| pos_5589 | Ferulate | down | down | up |
| pos_5590 | L-Rhamnose | down | down | up |
| pos_5611 | Schisanwilsonin D | down | down | up |
| pos_5633 | Lampranthin II | down | down | up |
| pos_565 | (9Z,12Z,15Z,18Z)-Tetracosatetraenoyl-CoA | up | up | down |
| pos_5653 | 4'-Methoxyresveratrol | up | up | up |
| pos_5665 | 2R-hydroxy-9Z,12Z,15Z-octadecatrienoic acid | up | up | up |
| pos_5668 | Lipoxin B4 | up | up | up |
| pos_570 | Pyridostigmine cation | up | up | up |
| pos_5728 | Naringerin | down | down | up |
| pos_5730 | Enoxacin | down | down | up |
| pos_5802 | L-Glutamyl-L-leucine | up | up | up |
| pos_5806 | 3,6'-Disinapoyl sucrose | down | down | up |
| pos_5809 | Pelargonidin-3-O-(6''-O-malonyl)glucoside | down | down | up |
| pos_5814 | (S)-Chiral alcohol | down | down | down |
| pos_5820 | 3,3',5-Trihydroxy-4',7-dimethoxyflavanone | down | down | up |
| pos_5821 | Rutacridone | up | up | up |
| pos_5904 | L-Phosphinothricin | down | down | up |
| pos_5909 | Phenylpropiolic acid | down | down | up |
| pos_5950 | 17,18-dehydro-clavulone I | up | down | up |
| pos_6045 | 2-Pentylthiophene | down | down | up |
| pos_610 | Aclacinomycin A | down | up | down |
| pos_6115 | Cortisone acetate | up | down | down |
| pos_6276 | Linamarin | down | down | up |
| pos_6336 | Argininosuccinic acid | down | up | down |
| pos_6362 | D-Fucose | up | down | up |
| pos_6365 | 1D-1-Guanidino-1-deoxy-3-dehydro-scyllo-inositol | up | down | up |
| pos_6393 | 13S-Hydroperoxy-6Z,9Z,11E-octadecatrienoic acid | up | up | up |
| pos_6434 | Nodakenin | down | down | down |
| pos_6602 | 5-Hydroxytryptophol (5HTOL) | down | down | up |
| pos_6623 | 5-(6-Hydroxy-3,7-dimethyl-2,7-octadienyloxy)-7-methoxycoumarin | down | down | down |
| pos_6625 | 5-hydroxy-1-(4-hydroxyphenyl)-7-(4-hydroxy-3-methoxyphenyl)-3-heptanone | down | down | down |
| pos_6710 | Hercynine | up | up | up |
| pos_6715 | Isococculidine | up | up | up |
| pos_6804 | Doxorubicin | down | down | up |
| pos_6872 | Deacetylisoipecoside | up | up | up |
| pos_6891 | Salicyl alcohol | up | up | down |
| pos_6895 | R-(-)-Mandelic acid | up | up | down |
| pos_6906 | 2,5-dimethyl-7-hydroxy-chromone | up | up | down |
| pos_6909 | Diphenyl carbonate | up | up | down |
| pos_6910 | 2-METHOXYXANTHONE | up | up | down |
| pos_6912 | Dalbergichromene | up | up | down |
| pos_7076 | Methylergonovine | up | down | down |
| pos_7316 | 5-(3-Hydroxypropyl)-7-methoxy-2-(3',4'-methylenedioxyphenyl)benzofuran | down | down | up |
| pos_7337 | 6'''-Deamino-6'''-hydroxyparomomycin II | down | down | up |
| pos_745 | 3-Methyloxindole | up | up | up |
| pos_746 | L-Tyrosine methyl ester | up | up | up |
| pos_747 | 3-(2-(methylamino)ethyl)-1H-indol-5-ol | up | up | up |
| pos_7546 | Primary fluorescent chlorophyll catabolite | up | up | down |
| pos_7590 | 6,10,14-Trimethyl-5,9,13-pentadecatrien-2-one | up | down | down |
| pos_7591 | 2-Linoleoyl Glycerol | up | down | down |
| pos_7593 | Atractylochromene | up | down | down |
| pos_7619 | (1R,10aS)-1,4,10,10a-Tetrahydrophenazine-1-carboxylate | down | up | down |
| pos_7786 | Coproporphyrinogen III | down | down | up |
| pos_7865 | D-Urobilin | up | up | down |
| pos_7912 | (+)-cis-3,4-Dihydrophenanthrene-3,4-diol | down | down | up |
| pos_7920 | Arg Tyr Tyr Met | down | down | up |
| pos_7965 | 3-Indoleacetic Acid | up | up | up |
| pos_8070 | 1-(3,4-Dihydroxyphenyl)-1-decene-3,5-dione | up | up | down |
| pos_8384 | Soyasaponin beta-A | up | up | down |
| pos_8420 | (S)-Cheilanthifoline | up | up | down |
| pos_8433 | Jasminoside C | up | up | down |

**Table S2.**Sample sequencing data quality control data statistics

| Samples | Clean reads | Clean bases | GC Content | %≥Q30 | Mapped Reads |
| --- | --- | --- | --- | --- | --- |
| afCK1 | 23,296,180 | 6,973,609,116 | 44.55% | 94.82% | 42,538,443 (91.30%) |
| afCK2 | 24,863,616 | 7,441,540,734 | 44.60% | 95.04% | 45,322,825 (91.14%) |
| afCK3 | 29,579,454 | 8,850,834,812 | 44.60% | 95.73% | 54,331,696 (91.84%) |
| afHD1 | 21,169,186 | 6,337,935,330 | 43.92% | 96.11% | 40,030,356 (94.55%) |
| afHD2 | 21,372,335 | 6,398,870,184 | 43.95% | 95.48% | 40,471,277 (94.68%) |
| afHD3 | 27,126,216 | 8,116,506,916 | 44.11% | 94.69% | 50,738,340 (93.52%) |
| jshCK1 | 26,781,751 | 8,016,955,800 | 44.05% | 95.90% | 50,861,644 (94.96%) |
| jshCK2 | 23,046,006 | 6,898,763,774 | 44.10% | 95.20% | 43,607,386 (94.61%) |
| jshCK3 | 26,641,038 | 7,974,070,972 | 44.58% | 95.29% | 48,662,974 (91.33%) |
| jshHD1 | 26,866,140 | 8,041,462,126 | 44.51% | 95.03% | 49,256,206 (91.67%) |
| jshHD2 | 23,680,870 | 7,088,841,484 | 44.56% | 95.38% | 43,559,004 (91.97%) |
| jshHD3 | 26,967,401 | 8,068,892,110 | 44.56% | 95.46% | 49,382,301 (91.56%) |
| qyCK1 | 23,948,491 | 7,167,449,846 | 43.93% | 96.05% | 45,440,639 (94.87%) |
| qyCK2 | 19,745,227 | 5,908,926,110 | 43.97% | 96.16% | 37,488,644 (94.93%) |
| qyCK3 | 22,580,074 | 6,759,465,114 | 44.02% | 95.63% | 41,891,267 (92.76%) |
| qyHD1 | 25,118,841 | 7,519,423,646 | 43.97% | 95.36% | 47,438,022 (94.43%) |
| qyHD2 | 27,636,912 | 8,273,567,462 | 43.84% | 95.82% | 52,496,063 (94.97%) |
| qyHD3 | 26,872,760 | 8,044,916,438 | 43.81% | 95.36% | 50,874,848 (94.66%) |

**Table S3.** Regulation of 66 common DEGs in afCK vs afHD, jshCK vs jshHD, and qyCK vs qyHD

| #ID | gene name | afCK vs afHD | jshCK vs jshHD | qyCK vs qyHD |
| --- | --- | --- | --- | --- |
| Cg1g001650 | Cg1g001650 | down | down | up |
| Cg1g001670 | Cg1g001670 | down | down | down |
| Cg1g003830 | Cg1g003830 | down | down | down |
| Cg1g008060 | Cg1g008060 | down | down | up |
| Cg1g012620 | Cg1g012620 | down | down | down |
| Cg1g015970 | Cg1g015970 | down | up | up |
| Cg1g017710 | Cg1g017710 | down | down | down |
| Cg2g002000 | Cg2g002000 | up | up | up |
| Cg2g003100 | Cg2g003100 | up | up | up |
| Cg2g006280 | Cg2g006280 | down | down | down |
| Cg2g010670 | Cg2g010670 | up | up | up |
| Cg2g010850 | Cg2g010850 | up | up | up |
| Cg2g011120 | Cg2g011120 | up | up | up |
| Cg2g017550 | Cg2g017550 | down | up | down |
| Cg2g021700 | Cg2g021700 | up | up | up |
| Cg2g028850 | Cg2g028850 | up | up | down |
| Cg2g038160 | Cg2g038160 | down | down | down |
| Cg2g040770 | Cg2g040770 | down | down | down |
| Cg2g043390 | Cg2g043390 | down | down | down |
| Cg3g008670 | Cg3g008670 | down | down | down |
| Cg3g009920 | Cg3g009920 | down | up | up |
| Cg3g014770 | Cg3g014770 | down | up | down |
| Cg3g021260 | Cg3g021260 | up | up | up |
| Cg3g024480 | Cg3g024480 | down | down | down |
| Cg3g024680 | Cg3g024680 | down | down | down |
| Cg4g016320 | Cg4g016320 | down | down | up |
| Cg4g017110 | Cg4g017110 | down | down | down |
| Cg4g017260 | Cg4g017260 | down | down | down |
| Cg4g024840 | Cg4g024840 | down | down | down |
| Cg5g001030 | Cg5g001030 | up | down | down |
| Cg5g004910 | Cg5g004910 | up | up | up |
| Cg5g010010 | Cg5g010010 | up | up | up |
| Cg5g020690 | Cg5g020690 | down | down | up |
| Cg5g020710 | Cg5g020710 | down | down | up |
| Cg5g020750 | Cg5g020750 | down | down | up |
| Cg5g023940 | Cg5g023940 | up | up | up |
| Cg5g027500 | Cg5g027500 | down | down | down |
| Cg5g028900 | Cg5g028900 | up | up | up |
| Cg5g036910 | Cg5g036910 | up | up | up |
| Cg5g039240 | Cg5g039240 | up | up | up |
| Cg6g009720 | Cg6g009720 | down | up | down |
| Cg6g010190 | Cg6g010190 | down | down | down |
| Cg6g017590 | Cg6g017590 | down | down | down |
| Cg6g023470 | Cg6g023470 | down | down | down |
| Cg6g024560 | Cg6g024560 | up | up | up |
| Cg6g025170 | Cg6g025170 | down | down | down |
| Cg7g003610 | Cg7g003610 | up | up | up |
| Cg7g007040 | Cg7g007040 | down | down | down |
| Cg7g007890 | Cg7g007890 | up | up | up |
| Cg7g010440 | Cg7g010440 | down | down | down |
| Cg7g013230 | Cg7g013230 | down | down | down |
| Cg8g004560 | Cg8g004560 | down | down | down |
| Cg8g018830 | Cg8g018830 | up | up | up |
| Cg8g021600 | Cg8g021600 | up | up | down |
| Cg8g024330 | Cg8g024330 | down | down | down |
| Cg9g018290 | Cg9g018290 | up | up | up |
| Cg9g023850 | Cg9g023850 | up | up | down |
| Cg9g023940 | Cg9g023940 | up | up | up |
| Cg9g024960 | Cg9g024960 | up | up | down |
| CgUng003780 | CgUng003780 | up | up | up |
| CgUng015820 | CgUng015820 | up | up | up |
| NewGene_1897 | -- | up | down | up |
| NewGene_4225 | -- | up | down | down |
| NewGene_4405 | -- | up | up | up |
| NewGene_691 | -- | down | down | down |
| NewGene_848 | -- | down | down | down |

**Table S4**. Statistics of transcription factor results

| Family | afCK vs afHD | | | jshCK vs jshHD | | | qyCK vs qyHD | | |
| --- | --- | --- | --- | --- | --- | --- | --- | --- | --- |
|  | up | dwon | count | up | dwon | count | up | dwon | count |
| *AP2/ERF-AP2* | 0 | 3 | 3 | 0 | 0 | 0 | 0 | 0 | 0 |
| *AP2/ERF-ERF* | 3 | 23 | 26 | 11 | 2 | 13 | 1 | 1 | 2 |
| *AP2/ERF-RAV* | 0 | 2 | 2 | 0 | 2 | 2 | 0 | 0 | 0 |
| *B3* | 2 | 1 | 3 | 1 | 0 | 1 | 1 | 0 | 1 |
| *B3-ARF* | 2 | 1 | 3 | 0 | 0 | 0 | 0 | 0 | 0 |
| *BES1* | 0 | 1 | 1 | 0 | 1 | 1 | 0 | 0 | 0 |
| *bHLH* | 3 | 14 | 17 | 6 | 3 | 9 | 1 | 1 | 2 |
| *bZIP* | 3 | 1 | 4 | 0 | 0 | 0 | 0 | 1 | 1 |
| *C2C2-CO-like* | 2 | 3 | 5 | 0 | 2 | 2 | 0 | 0 | 0 |
| *C2C2-Dof* | 1 | 4 | 5 | 2 | 0 | 2 | 0 | 0 | 0 |
| *C2C2-GATA* | 1 | 5 | 6 | 0 | 1 | 1 | 0 | 0 | 0 |
| *C2C2-LSD* | 0 | 1 | 1 | 0 | 0 | 0 | 0 | 0 | 0 |
| *C2C2-YABBY* | 0 | 2 | 2 | 0 | 1 | 1 | 0 | 0 | 0 |
| *C2H2* | 2 | 7 | 9 | 1 | 4 | 5 | 2 | 0 | 2 |
| *C3H* | 1 | 3 | 4 | 0 | 0 | 0 | 0 | 0 | 0 |
| *CSD* | 0 | 1 | 1 | 0 | 0 | 0 | 0 | 0 | 0 |
| *DBB* | 0 | 4 | 4 | 1 | 0 | 1 | 0 | 0 | 0 |
| *EIL* | 0 | 1 | 1 | 0 | 0 | 0 | 0 | 0 | 0 |
| *E2F-DP* | 0 | 0 | 0 | 1 | 0 | 1 | 0 | 0 | 0 |
| *GARP-ARR-B* | 0 | 0 | 0 | 0 | 1 | 1 | 0 | 0 | 0 |
| *GARP-G2-like* | 0 | 2 | 2 | 1 | 2 | 3 | 2 | 0 | 2 |
| *GeBP* | 1 | 1 | 2 | 0 | 0 | 0 | 0 | 0 | 0 |
| *GRAS* | 2 | 4 | 6 | 3 | 1 | 4 | 2 | 0 | 2 |
| *GRF* | 1 | 1 | 2 | 0 | 1 | 1 | 0 | 0 | 0 |
| *HB-BELL* | 1 | 0 | 1 | 0 | 1 | 1 | 0 | 0 | 0 |
| *HB-HD-ZIP* | 6 | 4 | 10 | 1 | 1 | 2 | 1 | 1 | 2 |
| *HB-other* | 0 | 2 | 2 | 0 | 1 | 1 | 0 | 1 | 1 |
| *HB-WOX* | 0 | 1 | 1 | 1 | 1 | 2 | 0 | 0 | 0 |
| *HSF* | 1 | 3 | 4 | 0 | 1 | 1 | 0 | 0 | 0 |
| *LIM* | 0 | 2 | 2 | 0 | 0 | 0 | 0 | 0 | 0 |
| *LOB* | 2 | 5 | 7 | 2 | 2 | 4 | 1 | 0 | 1 |
| *MADS-MIKC* | 2 | 0 | 2 | 1 | 0 | 1 | 0 | 0 | 0 |
| *MADS-M-type* | 1 | 2 | 3 | 0 | 0 | 0 | 0 | 0 | 0 |
| *MYB* | 10 | 4 | 14 | 14 | 5 | 19 | 1 | 1 | 2 |
| *MYB-related* | 6 | 5 | 11 | 1 | 0 | 1 | 1 | 0 | 1 |
| *NAC* | 5 | 8 | 13 | 6 | 3 | 9 | 0 | 2 | 2 |
| *NF-X1* | 0 | 1 | 1 | 0 | 0 | 0 | 0 | 0 | 0 |
| *NF-YA* | 1 | 0 | 1 | 0 | 0 | 0 | 1 | 0 | 1 |
| *NF-YB* | 1 | 0 | 1 | 0 | 0 | 0 | 0 | 1 | 1 |
| *OFP* | 0 | 2 | 2 | 0 | 1 | 1 | 1 | 0 | 1 |
| *PLATZ* | 1 | 1 | 2 | 0 | 1 | 1 | 0 | 0 | 0 |
| *SBP* | 1 | 0 | 1 | 0 | 1 | 1 | 0 | 0 | 0 |
| *SRS* | 0 | 1 | 1 | 0 | 0 | 0 | 0 | 0 | 0 |
| *TCP* | 2 | 1 | 3 | 0 | 0 | 0 | 0 | 1 | 1 |
| *Tify* | 0 | 3 | 3 | 2 | 1 | 3 | 0 | 1 | 1 |
| *Trihelix* | 0 | 2 | 2 | 1 | 2 | 3 | 0 | 0 | 0 |
| *TUB* | 1 | 0 | 1 | 0 | 0 | 0 | 0 | 0 | 0 |
| *WRKY* | 7 | 10 | 17 | 4 | 0 | 4 | 3 | 1 | 4 |
